# Construction of Self-Assembly Based Tunable Absorber: Lightweight, Hydrophobic and Self-Cleaning Properties

**DOI:** 10.1007/s40820-023-01108-3

**Published:** 2023-05-28

**Authors:** Zehua Zhou, Qianqian Zhu, Yue Liu, Yan Zhang, Zirui Jia, Guanglei Wu

**Affiliations:** 1 Institute of Materials for Energy and Environment, State Key Laboratory of Bio-fibers and Eco-textiles, College of Materials Science and Engineering, Qingdao University, Qingdao, 266071 People’s Republic of China; 2 College of Chemistry and Chemical Engineering, Qingdao University, Qingdao, 266071 People’s Republic of China

**Keywords:** MOF aerogel, Self-cleaning, Electromagnetic wave absorption, Hydrophobic

## Abstract

Although multifunctional aerogels are expected to be used in applications such as portable electronic devices, it is still a great challenge to confer multifunctionality to aerogels while maintaining their inherent microstructure. Herein, a simple method is proposed to prepare multifunctional NiCo/C aerogels with excellent electromagnetic wave absorption properties, superhydrophobicity, and self-cleaning by water-induced NiCo-MOF self-assembly. Specifically, the impedance matching of the three-dimensional (3D) structure and the interfacial polarization provided by CoNi/C as well as the defect-induced dipole polarization are the primary contributors to the broadband absorption. As a result, the prepared NiCo/C aerogels have a broadband width of 6.22 GHz at 1.9 mm. Due to the presence of hydrophobic functional groups, CoNi/C aerogels improve the stability in humid environments and obtain hydrophobicity with large contact angles > 140°. This multifunctional aerogel has promising applications in electromagnetic wave absorption, resistance to water or humid environments.

**Supplementary Information:**

The online version contains supplementary material available at 10.1007/s40820-023-01108-3.

## Highlights

- NiCo/C aerogel was prepared by pyrolysis carbonization self-assembly of NiCo- metal–organic frameworks (MOFs).
- The assembly mechanism of MOF aerogel was studied by adjusting the ratio of metal ion /BTC.
- Aerogel combines hydrophobic, lightweight, self-cleaning, and electromagnetic wave absorption properties.

**Supplementary Information:**

The online version contains supplementary material available at 10.1007/s40820-023-01108-3.

## Graphical abstract

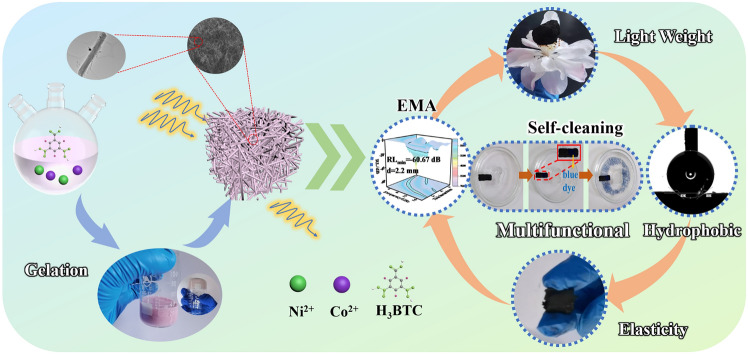

## Introduction

With the vigorous development of science and technology, a variety of electrical and electronic devices have emerged, especially the booming development of the fifth generation (5G) mobile technology operating at gigahertz electromagnetic frequencies, which has profoundly affected our lifestyles [1]. The emergence of electromagnetic wave hazards-related problems has many adverse effects on health as well as defense security, which also need to be urgently addressed [2–6]. As a result, there is a growing demand for strong absorption and durable microwave absorbers.

Typically, the prepared powdered absorbers require high filling levels, which lead to aggregation and high density [7]. Agglomeration behavior can be efficiently prevented if micro/nanocells are somehow ordered and uniformly grown in microscopic 3D nanofiber network [8, 9]. Accordingly, macroscopic 3D interconnected network is developed to lightweight EMA materials [10]. The characteristics of large surface area and high porosity can provide multiple interfaces and rich active sites for electromagnetic wave attenuation, increase the propagation path of electromagnetic wave, and achieve the purpose of improving impedance matching [11, 12]. Furthermore, ultra-low density of continuous 3D structure aerogel means extra-low filler load [13]. More importantly, in order to cope with harsh real-world applications, aerogels are required to be multi-functional [14, 15]. For example, aerogels with hydrophobicity and self-cleaning can flexibly cope with environments containing water [16].

Metal–organic frameworks (MOFs) are formed by combining transition metal ions and organic groups through covalent coordination bonds [17]. In the past decades, MOFs are usually used as ideal sacrificial templates for the preparation of various types of carbon-based nano-absorbing materials [18, 19]. However, the currently prepared MOF-derived carbon-based materials usually exist in the form of powder with low mechanical strength and low integrity [20], which often limits their application in practice and cannot be widely promoted [21]. To improve this drawback, MOF can be regarded as a self-assembled precursor, and its potential applications in wave absorption can be enhanced by expanding from simple conventional structures to complex structures at mesoscopic and macroscopic scales [22, 23]. The advantage is that it does not need to undergo complex chemical reactions, but is achieved by changing the physical structure only [24]. For example, Lohe et al. [25] earlier reported amorphous MOFs aerogels with micropores and macropores, which opened the way for the later synthesis of MOF aerogels. Zhang et al. [26] prepared MXene/RGO using magnetic nanochains as support. The excellent structural stability and strong mechanical properties ensure that it has the lowest reflection loss value based on the reported Mxene aerogels. Based on the structure of carbon fiber and the excellent thermal and compressive properties of polyimide rigid bubble walls and imide rings, Liu et al. [27] prepared ultra-wideband rigid porous foam absorbers up to 14 GHz (compressive strength up to 1.05 MPa).

In this work, we propose a simple strategy to prepare NiCo alloy nanoparticles embedded in carbon aerogel by water-induced NiCo-MOF self-assembly and pyrolytic carbonization under nitrogen atmosphere. It is extended from two-dimensional structure to 3D nanofiber structure. Due to the impedance matching of the 3D structure and the reasonable component design of CoNi/C, the ultralight NiCo/C aerogel can achieve high EMA performance with a maximum effective absorption bandwidth (EAB_max_) of 6.22 GHz. The constructed 3D network structure increases the EMA performance from the following aspects. (1) 3D structure of aerogel is beneficial to address the aggregation of nanoparticles, especially magnetic nanoparticles, resulting in high EMA performance at ultra-low loading (0.15 wt%). (2) The impedance matching of the 3D structure, the multi heterogeneous interface, and the defect-induced dipole polarization confer excellent EMA properties on CoNi/C aerogels. (3) The combination of hydrophobicity, self-cleaning, elasticity, and excellent EMA without changing the 3D structure suggests that CoNi/C aerogels are promising for applications such as resistance to water or humid environments.

## Experimental Section

### Materials

1,3,5-Benzenetricarboxylic acid (H_3_BTC), N, N-dimethylformamide (DMF), nickel nitrate hexahydrate (Ni (NO_3_)_2_.6H_2_O), cobalt nitrate hexahydrate (Co (NO_3_)_2_.6H_2_O), anhydrous ethanol and glycerol were bought from Aladdin. All chemicals were of analytical grade (AR) and used without further purification. The water was ultrapure water (18.25 MΩ cm).

Monometallic aerogels were prepared by replacing NiCo-MOF with Ni-MOF-1, Ni-MOF-2, Ni-MOF-3, Ni-MOF-4, Co-MOF-1, Co-MOF-2, Co-MOF-3, Ni-MOF-4, and other conditions remained unchanged (freeze drying). They were named as NCA-1, NCA-2, NCA-3, NCA-4, CCA-1, CCA-2, CCA-3, CCA-4, correspondingly.

### Characterization

The microscopic morphology and elemental distribution of the samples were analyzed using field emission scanning electron microscopy (SEM, JEOLJSM-7800F). The lattices of the samples were analyzed using transmission electron microscopy (TEM, JEOLJEM-2100). The Raman spectra of the samples were collected using a Raman spectroscopy system with a 50 m W DPSS laser at 532 nm. The molecular structures of the samples were obtained by powder x-ray diffraction (XRD, Rigaku Ultima IV with Cu–Ka radiation (λ = 0.15418)). The states of the elements of the samples were obtained by x-ray photoelectron spectroscopy (XPS) at 250Xi spectrometer and aluminum Kα-ray source. The molecular structures and chemical compositions of the samples were obtained by Fourier transform infrared spectroscopy (FT-IR, NicoletiS50) analysis. Hydrophobicity of the samples was measured by a contact angle measuring instrument (BOEN-6489). The thermogravimetric analysis (TGA) of the samples was recorded by an SDTQ600 analyzer. The electromagnetic parameters of each sample at test frequencies from 2 to 18 GHz were determined using a vector network analyzer (Agilent N5234A, USA) using the coaxial method, and the EMA characteristics of the samples were further calculated. The samples and paraffin wax were molten and mixed well by different mass ratio (The ratio of the mass of the sample to the mass of paraffin is defined as the loading), and the mixture was put into a grinding tool and pressed into a ring with an inner diameter of 3.04 mm and an outer diameter of 7 mm. The reflection loss (RL) value can be calculated based on transmission line theory. The formula is as follows [28–30]:

1 $$Z_{{{\text{in}}}} = Z_{0} \sqrt {\frac{{\mu_{r} }}{{\varepsilon_{r} }}} \tan \;h\left| {j\left( {\frac{2\pi fd}{c}} \right)\sqrt {\varepsilon_{r} \mu_{r} } } \right|$$

2 $${\text{RL}}({\text{dB}}) = 20\lg \left| {\frac{{Z_{{{\text{in}}}} - Z_{0} }}{{Z_{{{\text{in}}}} + Z_{0} }}} \right|$$

where *Z*_in_ is the impedance of the standard absorber, Z_0_ is the impedance in free space, *f* is the frequency of the incident electromagnetic wave, *d* is the thickness of the absorber layer, and *c* is the propagation speed of the electromagnetic wave in free space.

## Results and Discussion

### Composition and Structure

**Fig. 1:**
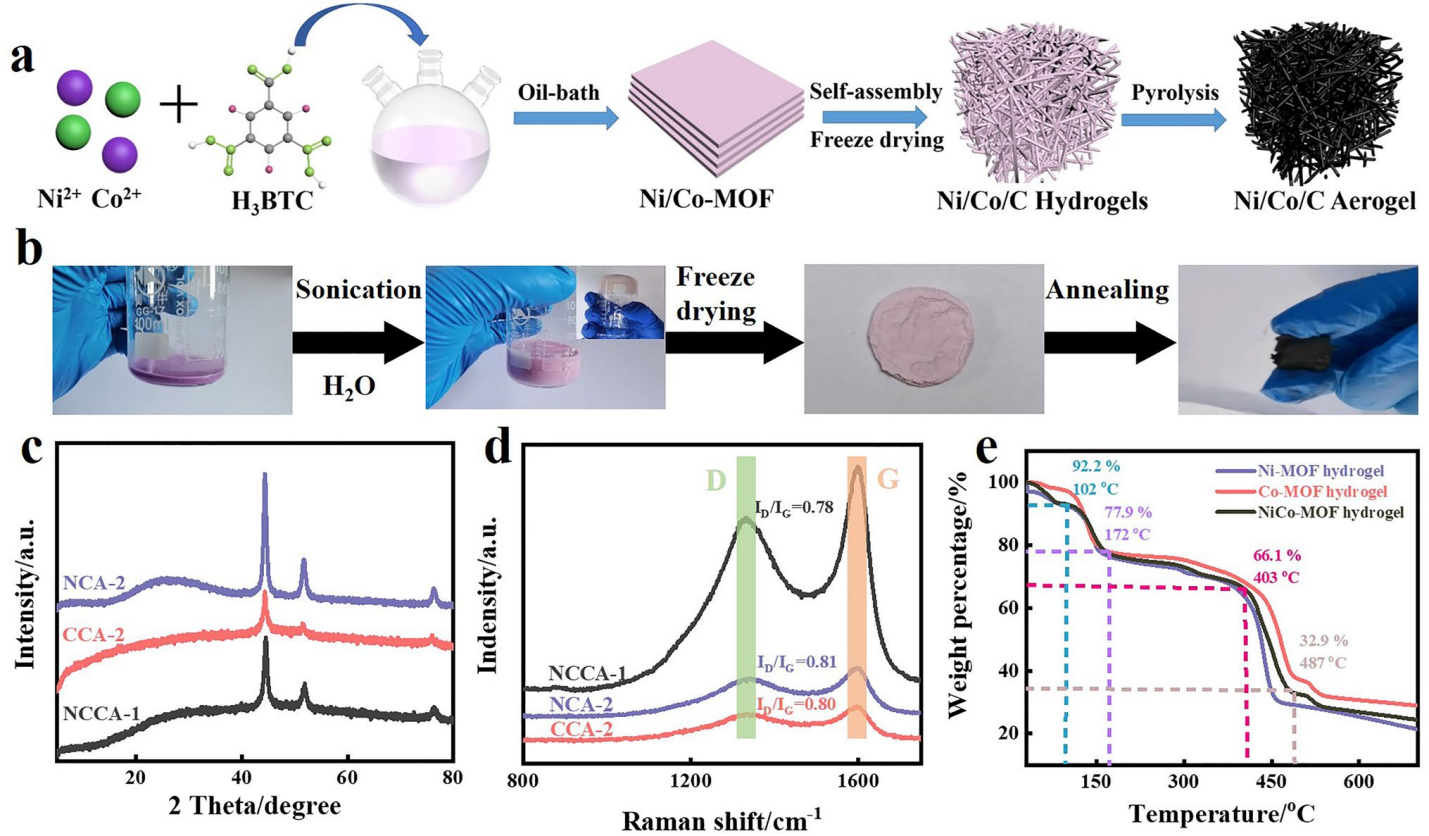
**a** Schematic illustration of the preparation process of NCCA-1. **b** NiCo/C hydrogel self-assembly process. **c** XRD pattern, **d** Raman spectra of NCA-2, CCA-2, NCCA-1. **e** TGA analysis of Ni-MOF hydrogel, Co-MOF hydrogel and NiCo-MOF hydrogel after freeze-drying

Since the synthesized NiCo-MOF is extremely sensitive to water, here we propose a facile strategy for the preparation of NiCo-MOF hydrogels by water-induced self-assembly. The preparation process of NCCA-1 is shown in Fig. 1a. NiCo-MOF was synthesized by oil bath method with Ni^2+^, Co^2+^ as metal ions and H_3_BTC as organic ligands [31]. NiCo-MOF was sonicated in a 1:9 solution of ethanol and water for several minutes. Turn the beaker upside down, the hydrogel can adhere to the beaker wall stably without falling off, indicating successful gelation (Fig. 1b). After that, the NiCo/C aerogel was synthesized by freeze-drying and calcination at 700 °C. The mechanism of aerogel assembly is as follows: (a) The laminar structure dissociates as water replaces DMF in the MOF structure; (b) The water molecule H combines with the O in the organic ligand in a form of hydrogen bonding, resulting in a partial dissolution of the organic ligand and metal ions in solution; (c) The metal center binds to the O of the water molecule, resulting in the assembly of the MOF; Hydrogen bonds formed between the H of the water molecule and the O in the organic ligand result in the directed assembly of Ni-MOFs [32]. Compared with the conventional aerogel preparation process, the water-induced self-assembly NiCo-MOF has the advantages of simple preparation, environmental protection, economy, and can be produced on a large scale.

The XRD spectra in Fig. 1c show the phase information and the crystal structure of NCA-2, CCA-2, NCCA-1. Three very distinct peaks can be observed, located at 76.4°, 51.8° and 44.5°, corresponding to the (220), (200) and (111) crystallographic planes of metallic Ni [32]. It is noteworthy that the diffraction peaks of CCA-2 and NCCA-1 overlap with NCA-2 due to the small difference between the diffraction peaks of Ni and Co [33]. In addition, the weak peak at 26.6° belongs to amorphous carbon [34], and there is no other characteristic peak, which proves that metal ions are successfully reduced to metal nanoparticles and graphitized carbon is formed by high temperature pyrolysis.

The Raman spectra of NCA-2, CCA-2, NCCA-1 are shown in Fig. 1d. The peak marked in light green, located at 1423.3 cm^−1^ is geared to the D peak and the peak marked in light orange, located at 1588.4 cm^−1^ belongs to the G peak. Usually, the values of *I*_*D*_/*I*_*G*_ are related to the level of graphitized carbon content and is inversely proportional. The values of *I*_D_/*I*_G_ for NCA-2, CCA-2, and NCCA-1 are 0.81, 0.80, and 0.78, respectively. The value of *I*_D_/*I*_G_ for NCCA-1 is the lowest, indicating that during the synthesis of aerogel, the amorphous carbon gradually decreases and more graphitized carbon is formed, which is beneficial to increase the electrical conductivity of the material and creating more conduction loss. The above results are consistent with the ratio of carbon diffraction peak intensity to metal (metal alloy) diffraction peak intensity in XRD (Fig. S2b). In addition, this also explains why the carbon peak intensity of NCA-2 in XRD is greater than that of NCCA-1, which cannot directly reflect the degree of graphitization.

Thermogravimetric analysis was performed for Ni-MOF hydrogel, Co-MOF hydrogel and NiCo-MOF hydrogel. As shown in Fig. 1e, we can find four decreasing phases in the curves. In the first stage, the slight decrease of 7.8% in the curve before 102 °C is attributed to the evaporation of water from the surface of material. In the second stage, in the temperature range of 102–172 °C, the mass decreases by 14.3 wt%, which is attributed to the evaporation of crystalline water from the lattice. In the third stage, in the temperature range of 171–403 °C, the mass decreases by 11.8 wt%, which is attributed to the volatilization of residual solvent in the NiCo-MOF hydrogel [32]. The fourth stage, which decreases sharply in the temperature range of 403–487 °C with a mass decrease of about 33.2 wt%, is attributed to the decomposition and carbonization of organic matter in the hydrogel to form pyrolytic carbon, which reduces metal ions to metal nanoparticles dispersed in the carbon layer. When the temperature continued to increase, the curve hardly changed significantly, indicating that no thermochemical reaction occurred. Similar alterations may be seen in the Ni-MOF hydrogel and Co-MOF hydrogel TGA curves. The final residual metal content is in the order of Co-MOF hydrogel > NiCo-MOF hydrogel > Ni-MOF hydrogel. This is consistent with the relative molecular mass size of the elements Co and Ni. Therefore, it is not difficult to see that NCCA-1 exists in the form of NiCo alloy combined with XRD pattern (Fig. 1a). Compared with elemental metals, the alloy can change the distribution of charge, induce the center of polarization, orient the internal electrons and convert electromagnetic energy into heat energy under the action of induced electric field.

**Fig. 2:**
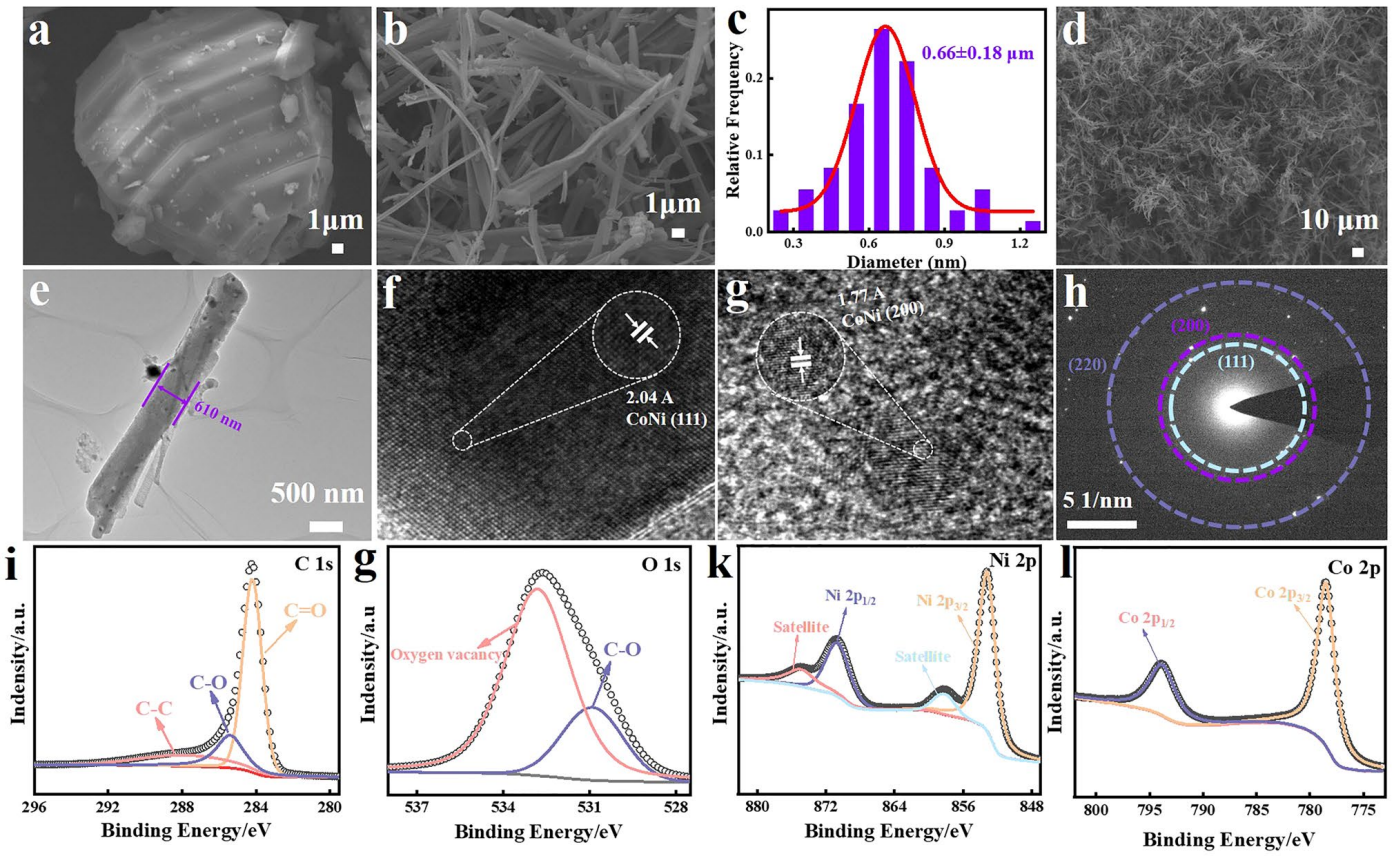
**a** SEM images of NiCo-MOF. **b, d** SEM images of NCCA-1 at different magnifications. **c** diameter distribution, **e** TEM, **f, g** HR-TEM and **h** SAED images of NCCA-1. **i** C 1*s* spectra, **g** O 1*s* spectra, **k** Ni 2*p* spectra and **l** Co 2*p* spectra of the NCCA-1

To investigate the morphological evolution during aerogel synthesis, NiCo-MOF and NCCA-1 morphologies were characterized by SEM. NiCo-MOF morphology is a bulk structure consisting of lamellar structures with a thickness of 25–35 μm (Fig. 2a), and Ni-MOF-2 and Co-MOF-2 also exhibit the same lamellar structure (Fig. S5a, d), indicating that changing the metal nitrate does not change the morphology of MOF. This lamellar structure under the strong action of ultrasound undergoes more easily peeled off from each other along the radial direction to form a dispersed 3D fibrous network structure, which in turn forms a hydrogel by self-assembly under induction of water. Figure 2b, c shows the scans of NCCA-1 at different magnifications. It can be seen that the morphology of the carbonized aerogel is a nanofibrous structure with smooth surface and uniform dispersed phase. The diameter distribution in Fig. 2d shows that the diameter of the fibers is about 0.48–0.84 μm. Figure 2e shows the TEM image of a single nanofiber in NCCA-1, which has a slender rod-like structure and smooth surface. By calculating that its diameter is 610 nm, the results are consistent with those in Fig. 2c. The diameter of NiCo alloy nanoparticles is between 15 and 25 nm (Fig. S1). The plane spacing of CoNi (111) crystal planes (2.04 Å) can be observed in Fig. 2f. Similarly, the planar spacing of 1.77 Å are corresponding to (200) crystalline plane, respectively (Fig. 2g). As can be seen from the EDS mapping, Ni, O, C elements are present in NCA-2 (Fig. S10); and Co, O, C elements in CCA-2 (Fig. S11); and Ni, Co, O, C elements in NCCA-1 (Fig. S9), respectively. It further illustrates the high homogeneity and dispersion of NiCo alloy particles on the surface. Selected Area Electron Diffraction (SAED) enables further analysis of the phase information of NCC-1. The three diffraction rings as shown in Fig. 2h correspond to the (111), (200), and (220) crystal planes of the NiCo alloy, demonstrating the successful synthesis of NCCA-1.

The electronic states and the types of elements on the surface of aerogel were further investigated using XPS spectra. In Fig. S15, the XPS spectrum of NCCA-1 shows characteristic peaks at 284°, 533°, 778°, and 854° corresponding to C 1*s*, O 1*s*, Co 2*p*, and Ni 2*p*, respectively. The C 1*s* spectra of NCCA-1 can be divided into three peaks (Fig. 2i) at 284.2, 285.4, 286.3 eV, corresponding to C–C, C–O, C=O [35]. The O 1*s* spectra of NCCA-1 is shown in Fig. 2g, and two distinctive peaks can be observed, the one at 530.9 eV corresponding to C–O and the one at 532.8 eV indicating the presence of oxygen vacancies [36]. Oxygen vacancies can change the charge transfer capability within the material and alter the conductivity, which can facilitate the absorption of electromagnetic waves. As seen in the Ni 2*p* XPS spectra (Fig. 2k), the characteristic peaks of Ni 2*p*_1/2_ and Ni 2*p*_3/2_ are located near 870.6 and 853.2 eV [37] and the two weak peaks near 874.9 eV and 858.2 eV belong to the satellite peaks of Ni 2*p*_1/2_ and Ni 2*p*_3/2_. As seen in the Co 2*p* XPS spectra (Fig. 2l) near 778.5 and 793.8 eV attributed to Co 2*p*_3/2_ and Co 2*p*_1/2_ [38]. Meanwhile, another deconvolution peak at 780.5 eV may belong to Co (II), which may be caused by partial surface oxidation of metallic cobalt. In addition, the XPS spectra of NCA-2 and CCA-2 coincide with those of NCCA-1, further suggesting that the generation of NiCo alloys in NCCA-1 (Figs. S16 and S17).

### Electromagnetic Performance and Mechanism

**Fig. 3:**
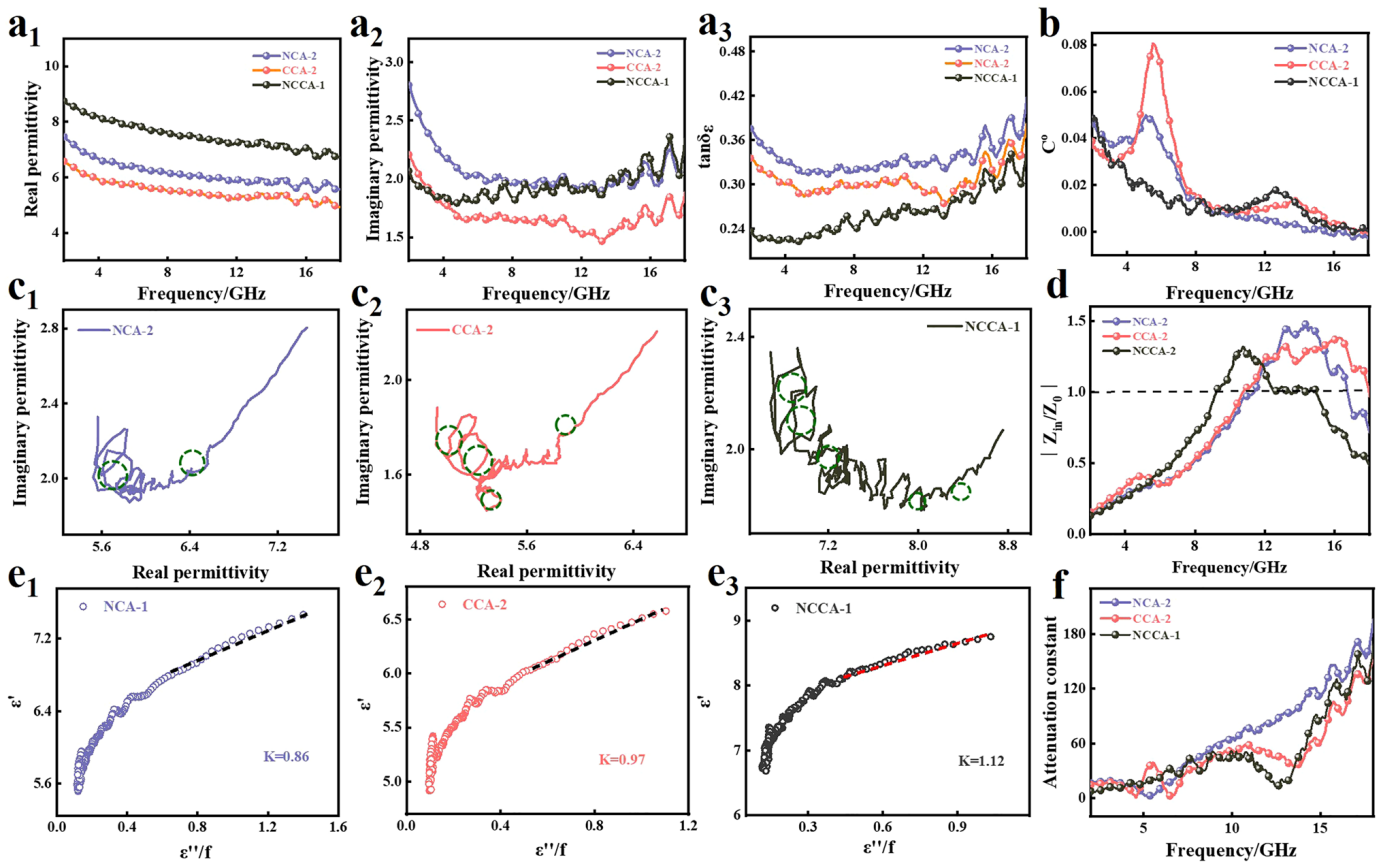
**a**_**1**_ Permittivity real part, **a**_**2**_ imaginary part, **a**_**3**_ tanδ_ε_ values of NCA-2, CCA-2, NCCA-1. Cole–Cole of **c**_**1**_ NCA-2, **c**_**2**_ CCA-2, **c**_**3**_ NCCA-1. The curves of ε′ versus ε″/f of **e**_**1**_ NCA-1, **e**_**2**_ CCA-1, **e**_**3**_ NCCA-1. **b** C_0_, **d** impedance matching, **f** attenuation constant of NCA-2, CCA-2, NCCA-1

Figures 3a_1_, a_2_ and S18a, b show the complex permittivity (ε_r_ = ε′ − jε″) and complex permeability (μ_r_ = μ′ − jμ″) of NCA-2, CCA-2, NCCA-1 in 2–18 GHz [39–41]. The ε′ values of the three samples show a decreasing trend, which is caused by the Debye polarization behavior of the dipole lagging behind the change of electromagnetic wave incident frequency [42]. The values of ε′ decrease from 7.5, 6.5, 8.7 to 5.5, 4.9, 6.6, respectively. ε′ value of NCCA-1 is much larger than NCA-1 and CCA-1, indicating its stronger electromagnetic wave storage capacity. The ε″ curve also tends to decrease with increasing frequency and is accompanied by multiple resonance peaks (Fig. 3a_2_), which is evidence of dielectric loss formation. It indicates that the introduction of metal nanoparticles increases the density of interfacial contacts, which can generate more interfacial polarization. When the frequency is increased to the range of 13–18 GHz, there is a slight increase in the value of ε″ and several distinct relaxation peaks, which are the result of the dipole reorientation with increasing electric field frequency [43] and the presence of multiple polarization relaxation processes [44, 45]. NCCA-1 does not have the highest ε″ value in the low frequency region (2–10 GHz) compared to NCA-2 and CCA-2, which is the reason for the unsatisfactory value of Tanδ_ε_ (Tanδ_ε_ = ε′/ε″) (Fig.  3a_3_). However, the final wave absorption performance of NCCA-1 is the best, which is due to the poor impedance matching (Fig. 3d) and low ε′ values of NCA-2 and CCA-2, resulting in the electromagnetic waves not being reflected well into the interior of the material. This will be described below.

Cole–Cole curves can reflect the polarization process inside the material. The mechanism of dielectric loss is revealed by the Debye dipole relaxation process, which can be described by Eq. 3 [46, 47]:

3 $$\left( {\varepsilon{^{\prime}} - \frac{{\varepsilon_{{\text{s}}} + \varepsilon_{\infty } }}{2}} \right) + \left( {\varepsilon{^{\prime\prime}} } \right)^{2} = \left( {\frac{{\varepsilon_{{\text{s}}} - \varepsilon_{\infty } }}{2}} \right)^{2}$$

where *ε*_*s*_ is the static permittivity and *ε*_*∞*_ is the relative permittivity at infinite frequency.

According to Fig. 3c_1_–c_3_, there are several Cole–Cole semicircles in NCA-2, CCA-2, NCCA-1, indicating that the existence of polarization sites can cause the change of surrounding charge potential and form polarization relaxation [48]. Compared with NCA-2, CCA-2, the Cole–Cole semicircles of NCCA-1 not only dominate in number, but also have more regular radius, indicating the existence of more dielectric loss mechanisms to attenuate the electromagnetic wave (e.g., dipole polarization from oxygen vacancies and interfacial polarization between multi-component heterogeneous interfaces). The advantage in the number of semicircles may be related to the synergistic effect of NiCo bimetallic alloy nanoparticles, which are able to provide multiple types and numbers of dipoles and multiple heterogeneous interfaces and defects to form more relaxation processes. The evidence that conduction losses play a large role in the consumption of electromagnetic waves in comes from the near-linear tails on the Cole–Cole plots of NCA-2 and CCA-2 [49]. The relaxation time can be expressed by the Eq. 4:

4 $$\varepsilon{^{\prime}} = \frac{1}{2\pi \tau }\frac{{\varepsilon{^{\prime\prime}} }}{f} + \varepsilon_{\infty }$$

According to Eq. 4, the relationship between ε′ and ε″/f is linear when the electric dipole polarization is the only way to dielectric loss. It is noteworthy that the relationship is linear at 8–18 GHz and nonlinear at 2–8 GHz for all samples, demonstrating the simultaneous existence of dipole polarization and interfacial polarization. Polarization relaxation time can be determined by the ratio of ε′ versus ε″/f. As shown in Fig. 3e_1_–e_3_, the relaxation times of NCA-2, CCA-2 and NCCA-1 are 0.86, 0.97, 1.12, respectively. The relaxation time of different samples may be different for the following reasons: (a) The electric field environment around dipoles is not the same, so the orientation of dipoles is uncertain; (b) The two types of polarization relaxation correspond to different polarization relaxation times, and thus have different attenuation abilities to electromagnetic waves [36, 50].

Figure S18a, b shows the μ′ and μ″ images of the three samples, respectively. Among them, the curves of μ′ and μ″ of the three samples show several fluctuations of different magnitudes in the range of 4–7 GHz (low frequency) and 12–14 GHz (high frequency), indicating the existence of exchange resonance, eddy current effect, and natural resonance multiple magnetic loss mechanisms [50]. Usually, we consider that the C_0_ values remain constant over a certain frequency range when the cause of magnetic loss is mainly Eddy current resonance, and the C_0_ values vary drastically when the cause of magnetic loss is mainly exchange resonance. C_0_ values can be expressed by Eq. 5 [51]:

5 $$C_{0} = \mu{^{\prime\prime}} \left( {\mu{^{\prime}} } \right)^{ - 2} f^{ - 1} = 2\pi \mu_{0} d^{2} \delta$$

From Fig. 3b, it can be seen that the C_0_ value varies drastically with frequency at low frequencies from 2 to 8 GHz. And the fluctuation is attributed to exchange resonance. At high frequencies from 13 to 18 GHz, the fluctuation caused by eddy current loss can be verified by the constant relationship between C_0_ value and frequency [52]. Therefore, it can be concluded that exchange resonance and natural resonance are the main sources of magnetic loss.

Figures 3a_3_ and S18c show the tanδ_ε_ and Tanδ_μ_ curves of NCA-2, CCA-2, and NCCA-1. In general, the dielectric and magnetic losses of NCCA-1 are better than NCA-2 and CCA-2. Therefore, NCCA-1 has satisfactory EMA performance. Relative to the weak reaction of magnetic loss, dielectric loss is the dominant factor of electromagnetic loss.

The impedance match (Z) and the attenuation constant (α) are two other important indicators of EMA performance [53, 54]. When Z is close to 1, the electromagnetic waves are maximized to be incident inside instead of being reflected out. A larger α value represents a stronger ability to attenuate electromagnetic waves. We studied the normalized input impedance (Fig. 3d) at the thickness of 2.2 mm. The impedance matching of NCA-2, CCA-2 is poor, while NCCA-1 can maintain good impedance matching in a wide frequency range (12–15 GHz) with Z≈1. According to Maxwell–Garnett theory, the 3D structure facilitates impedance matching optimization, allowing more electromagnetic waves to enter the material. 3D nanofiber networks shape high-density 3D conductive networks, effectively expand the effective absorption bandwidth of NCCA-1, and obtain satisfactory dielectric parameters. Figure S33 gives the variation of Z with frequency at the matched thickness of 1.5–3.0 mm. As the matched thickness increases, the value of the *RL*_min_ shifts toward lower frequencies. When the matched thickness is 2.2 mm, the impedance match of the sample is close to 1 and has the optimal absorption performance.

The attenuation constant α is one of the indexes to measure the attenuation ability of the absorber to electromagnetic waves and can be calculated by the Eq. 6 [55, 56]:

6 $$\alpha = \frac{\sqrt 2 \pi f}{c}\sqrt {\left( {\mu^{^{\prime\prime}} \varepsilon^{^{\prime\prime}} - \mu^{^{\prime}} \varepsilon^{^{\prime}} } \right) + \sqrt {\left( {\mu^{^{\prime\prime}} \varepsilon^{^{\prime\prime}} - \mu^{^{\prime}} \varepsilon^{\prime}} \right)^{2} + \left( {\mu^{\prime}\varepsilon^{\prime\prime} - \mu^{\prime\prime}\varepsilon^{\prime}} \right)^{2} } }$$

where *c* denotes the speed of propagation of light in free space and *f* denotes the frequency.

As shown in Fig. 3f, the α values of NCA-2, CCA-2, and NCCA-1 reached 196.19, 161.57 and 168.86, respectively. And the attenuation coefficients of NCCA-1 and NCA-2 are relatively good. The factors that determine the wave absorbing performance of the absorber are multiple. Due to the poor impedance matching of NCA-2, the absorbing performance is still unsatisfactory, although its attenuation coefficient value is high.

**Fig. 4:**
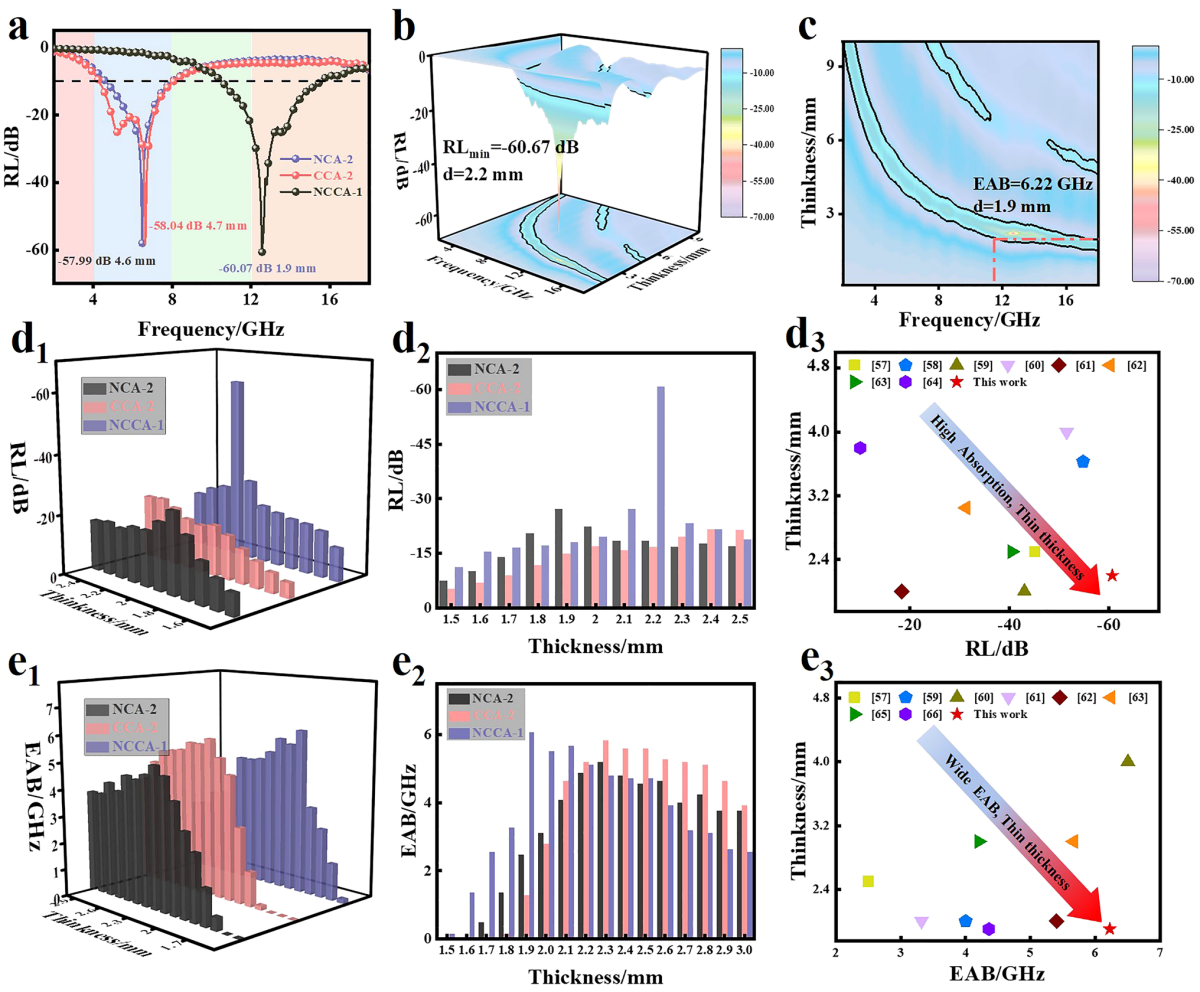
**a** RL and corresponding matching thickness of NCA-2, CCA-2, NCCA-1. **b** 3D RL diagram and **c** 2D RL diagram of NCCA-1. **d**_**1**_ 3D and **d**_**2**_ 2D RL diagrams of NCA-2, CCA-2, NCCA-1 at 1.5–2.5 mm. **d**_**3**_ The RL comparison between previous works and this work. **e**_**1**_ 3D and **e**_**2**_ 2D EAB diagrams of NCA-2, CCA-2, NCCA-1 at 1.5–3.0 mm. **e**_**3**_ The EAB comparison between previous works and this work

To further analyze the EMA characteristics of NCA-2, CCA-2, NCCA-1, we analyzed their 3D and 2D EMA performance curves at different thicknesses. As shown in Fig. 4a, the *RL*_min_ of NCCA-1 is − 60.67 dB at 2.2 mm (Fig. 4b); while the RL_min_ of NCA-2 is − 57.99 dB at 4.6 mm (Fig. S25a), and the RL_min_ of CCA-2 is − 58.04 dB at 4.7 mm (Fig. S25b). The matching thickness of NCA-2 and CCA-2 is thicker relative to NCCA-1. Therefore, NCA-2 and CCA-2 cannot meet the requirement of "thin" in practical applications, which will be limited in practical applications. The EAB is usually regarded as a very important indicator of the absorber. The 2D EMA performance curve (Figs. 4c and S26) can intuitively reflect the EAB of the three samples. At the thickness of 2.3, 2.3, and 1.9 mm, the EAB_max_ of NCA-2, CCA-2, and NCCA-1 are 5.20, 5.84, and 6.22 GHz, respectively The EAB of NCCA-1 is the largest, indicating that 90% absorption of electromagnetic waves in a wider frequency range can be achieved at a very thin thickness.

Figure 4d_1_, d_2_, e_1_, e_2_ depict the 3D and 2D bar plots of RL and EAB for the three samples at thicknesses of 1.5–2.5 and 1.5–3 mm. From the column distribution plots, it is more intuitive that NCCA-1 has ideal RL and EAB values, indicating that it can be used as an ideal absorber. As a result, the electromagnetic wave is propelled by the charge to create a high density vortex due to the synergistic impact of the metal alloy and the optimization of the permeability. The relatively unstable lattice of the alloy is more likely to produce more lattice distortion, lattice strip loss, vacancy and defect, forming the center of polarization, leading to more relaxation loss. Figure 4d_3_, e_3_ compare the present work with the published work related to EMA aerogels. It can be found that our prepared NCCA-1 is outstanding in both *RL*_min_ [57–64] and EAB [57, 59–63, 65, 66], indicating that NCCA-1 can be used as a potential absorber. A large number of uniformly dispersed metal alloy particles make up for the loss of magnetic and contribute significantly to the dielectric loss. The conductive network formed by 3D structure further increases the conductivity loss of the system. In addition, abundant heterogeneous interfaces are formed, resulting in a large number of defects. This gives NCCA-1 excellent dielectric and magnetic loss capabilities, which has great potential to be an ideal material compared to other electromagnetic wave aerogels. More important is the practical application. The preparation method for the MOF aerogel in this study is universal and has reference relevance for other MOF in addition to its great performance. Also, the preparation procedure is straightforward and affordable, opening the door to large-scale production. Remarkably, it can preserve a complete 3D structure compared to other minuscule rod-like materials. Also, unlike the layered structure, the odd rod-like structure may be changed to meet demand and various varieties of aerogel can be created to increase its functionality.

**Fig. 5:**
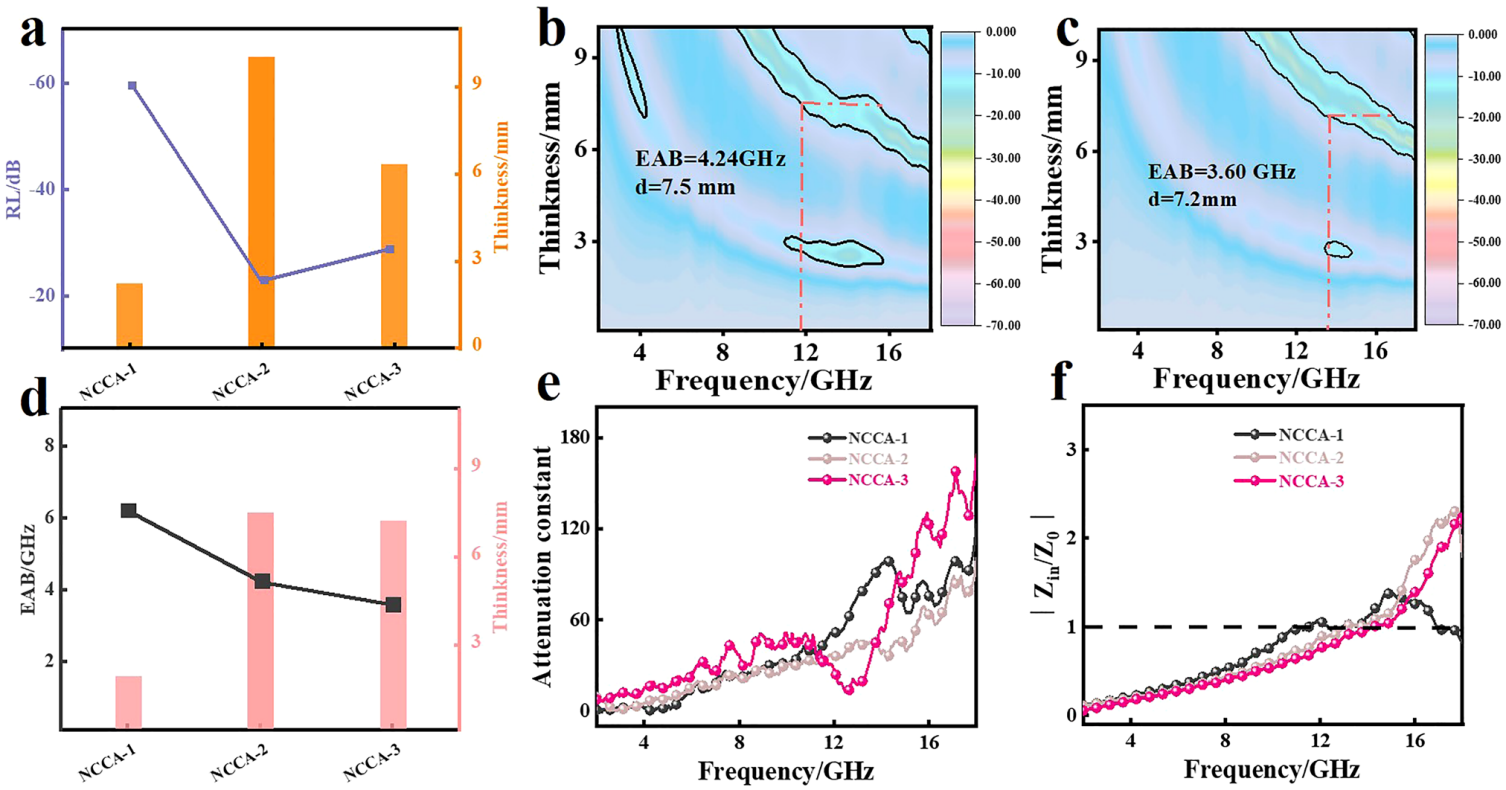
**a** RL and corresponding matching thickness of NCCA-1, NCCA-2, NCCA-3. **b**, **c** 2D RL diagram of NCCA-2, NCCA-3. **d** EAB and corresponding matching thickness of NCCA-1, NCCA-2, NCCA-3. **e** Attenuation constant and **f** impedance matching of NCCA-1, NCCA-2, NCCA-3

To verify the advantages of the 3D nanofiber structure formed by freeze-drying, we compared the wave absorption properties of NCCA-1 after freeze-drying, blast drying and vacuum drying. XRD and EDS showed that different drying methods had no effect on the final product (Figs. S2, S12 and S13). We compared the EAB and *RL*_min_ of NCCA-1, NCCA-2 and NCCA-3 and the corresponding matching thickness (Fig. 5a, d). As shown in Figs. 5b, c and S31, the *RL*_min_ of NCCA-3 is − 28.94 dB at 6.3 mm and the EAB is 3.6 GHz at 7.2 mm, the *RL*_min_ of NCCA-2 is − 22.47 dB at 10.0 mm and the EAB is 4.24 GHz at 7.5 mm. The samples obtained by blast drying and vacuum drying, both EAB, RL_min_, and the best-fit thickness are far inferior to those obtained by freeze-drying. This is due to the collapse of the 3D fiber network structure of NCCA-2 and NCCA-3 (Fig. S8), which further verifies the advantage of the 3D structure. In addition, the values of ε′, ε", Tanδ_ε_, μ′, μ", and Tanδ_μ_ (Fig. S21) of NCCA-1 are much larger than NCCA-2 and NCCA-3. NCCA-1 has more relaxation peaks, Col-Col semicircle (Figs. S24 and 3c_3_), and maximum attenuation constant (Fig. 5e). Therefore, the polarization relaxation is more pronounced and the attenuation of electromagnetic waves is stronger. In addition, the advantage in impedance matching is one of the key factors (Fig. 5f). Besides, it can be concluded that vacuum drying and blast drying cannot maintain the 3D network fiber structure of the aerogel, which leads to the collapse of the final sample micromorphology and thus the aggregation, and the electromagnetic waves cannot enter the material better and have a negative effect on the final wave absorption performance. On the contrary, the 3D structure network has the following benefits for absorbing electromagnetic wave: (a) promoting the absorption of penetrating electromagnetic wave through multiple reflections, effectively reducing the number of heat transfer paths in the aerogel; (b) greatly improving the conductive permeability threshold of the material and improving the efficiency of electron transmission; (c) optimizing the impedance matching.

**Fig. 6:**
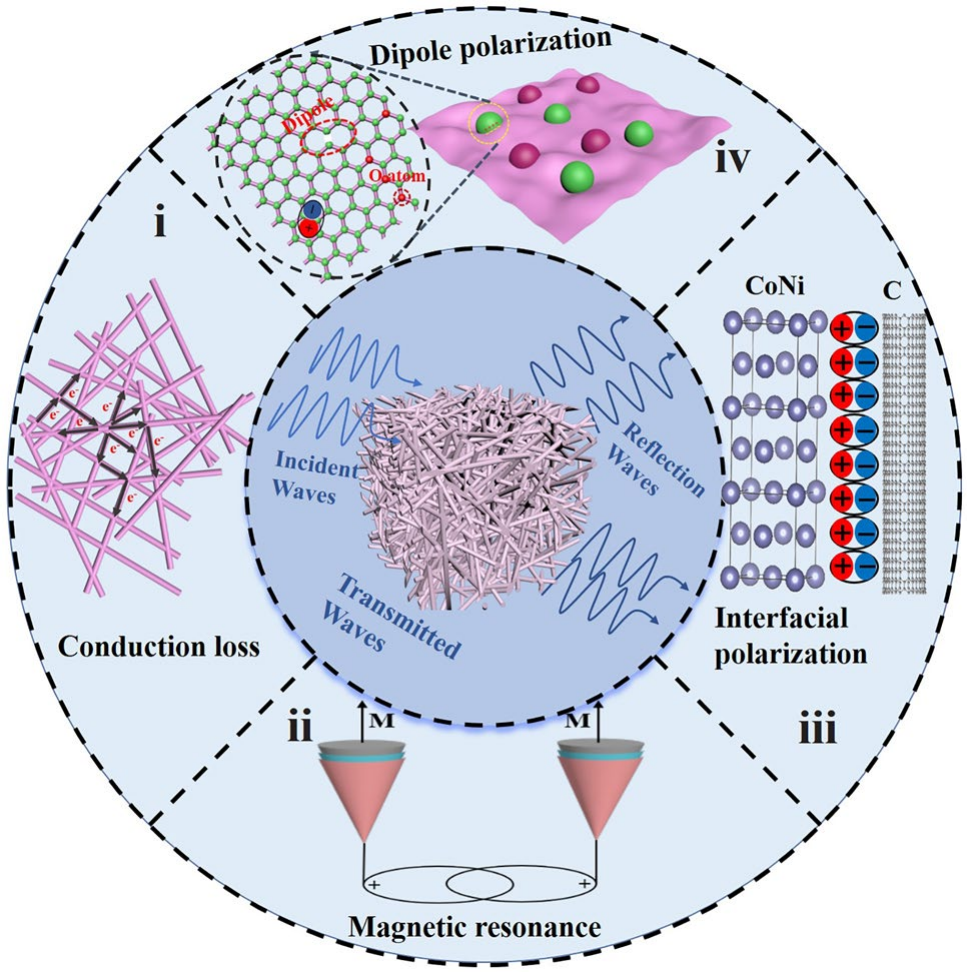
Schematic diagrams of the potential microwave absorption mechanisms of NCCA-1

Combined with the above analysis, the potential wave absorption mechanism of NCCA-1 aerogel is shown in Fig. 6:

(1) The impedance matching and attenuation constants allow more incident electromagnetic waves to enter the material and dissipate rather than be reflected [67, 68].

(2) The electromagnetic wave reflects and scatters back and forth in the gap on the material surface, which increases the propagation path and attenuates the electromagnetic wave [69, 70].

(3) The oxygen vacancies and defects formed during the high temperature carbonization process can form dipole polarization to dissipate electromagnetic waves [71, 72].

(4) The free electrons in graphite carbon leave their equilibrium positions under the action of an applied electromagnetic field, forming a microcurrent, resulting in conductive losses [73, 74].

(5) The introduction of magnetic nanoparticles can form, exchange resonance natural resonance and eddy current loss [75].

(6) High-density contact interface between NiCo nanoalloy and graphitic carbon layer triggers interfacial polarization.

### Superhydrophobic and Self-Cleaning Properties

**Fig. 7:**
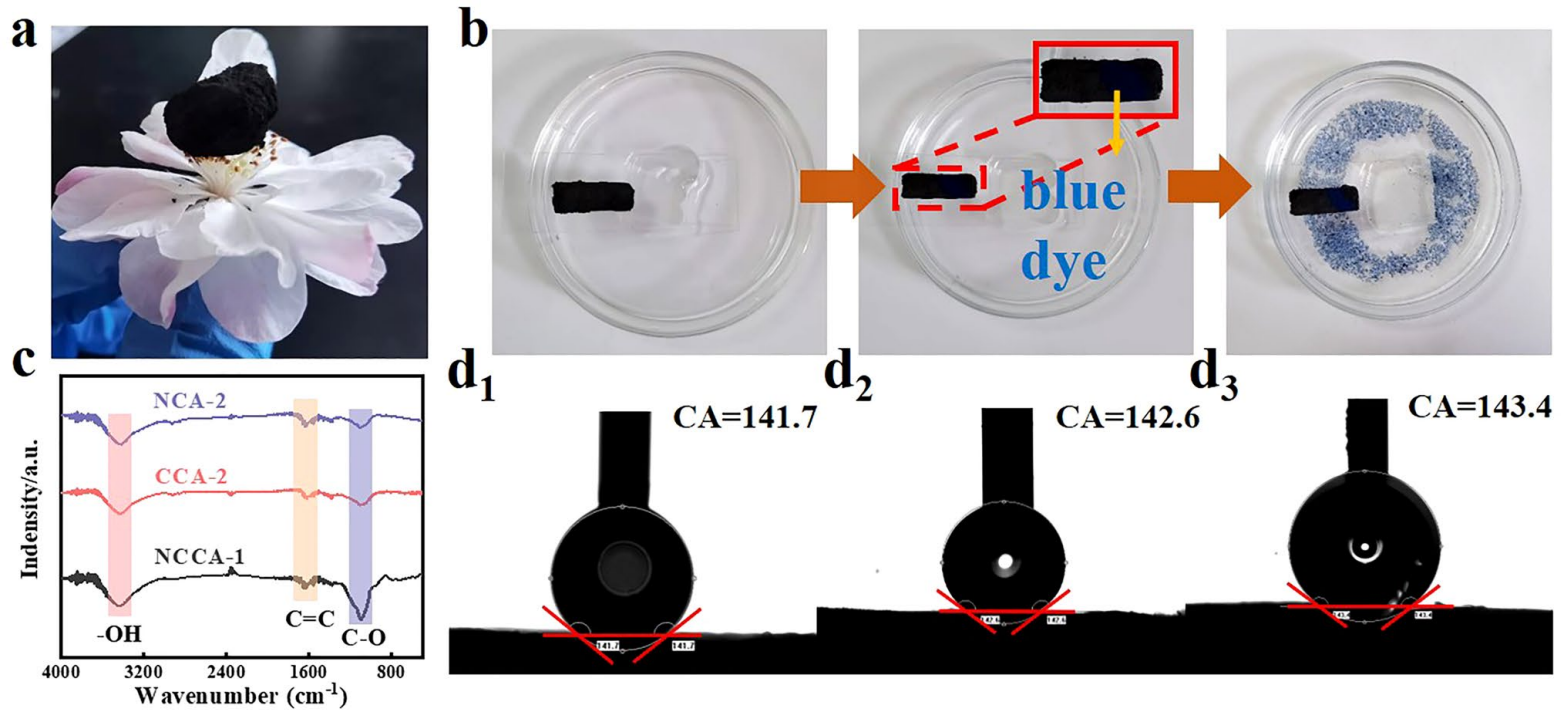
**a** Photographs of NCCA-1 on top of a flower. **b** Self-cleaning experiment process of NCCA-1. **c** FT-IR pattern of NCA-2, CCA-2, NCCA-1. Water contact angle of **d**_**1**_ NCA-2, **d**_**2**_ CCA-2, **d**_**3**_ NCCA-1

The aerogel has the property of low density due to the existence of enormous air-filled voids inside the aerogel [76]. To verify that the prepared aerogels have the attractive property of low density, we placed NCCA-1 on the blooming stamen (Fig. 7a), and NCCA-1 was able to make a stable stay on top of the flower without deforming the stamen. In addition, the prepared aerogels are hydrophobic. We measured the water contact angles of three samples. The maximum water contact angles of the three samples were 141.7, 142.6, and 143.4, respectively (Fig. 7d_1_–d_3_). The FT-IR spectra of NCA-2, CCA-2, and NCCA-1 are shown in Fig. 7c. The three samples show the same identical peak positions. The vibrational bands at 3434, 1612, and 1110 cm^−1^ correspond to the stretching vibrations of –OH, the aromatic C=C group and the C–O group [77, 78]. The sample does not contain hydrophilic functional groups after pyrolysis, which is one of the direct reasons for the hydrophobic properties of the aerogel. In addition, NCCA-1 has the highest peak intensity, indicating that it contains the highest concentration of hydrophobic functional groups, which directly proves that NCCA-1 is more hydrophobic than NCA-2 and CCA-2. The mechanism by which a hydrophobic group is hydrophobic may be as follows: Hydrophobic groups cannot hydrogen bond with water, so it will break the hydrogen bond network of water. The broken hydrogen bonds are driven to rearrange as much as possible along the oriented structure, and a water "cage" structure forms around the nonpolar surface. But this breakdown in hydrogen bonds can't fully compensate, resulting in an increase in the entropy of the water molecules. Therefore, the hydrophobic interaction induced by hydrophobic groups is the result of the entropy increase drive [79].

Due to the hydrophobicity of aerogel, it may have potential application in self-cleaning function [80]. Figure 7b tests the self-cleaning process of NCCA-1. NCCA-1 was placed on a clean slide, the surface of the aerogel was contaminated with blue dye, and then flushed with a steady stream of water. Water droplets slide on the surface of the aerogel and rinse the blue dye into the petri dish. When water comes into contact with the surface of the NCCA-1, a stable layer of air is formed, which keeps the droplets from penetrating into the aerogel [81]. In Fig. 7b, the blue dye floating in the water can be observed, indicating that the blue dye was successfully washed off the surface of the aerogel, which proves that the hydrophobic NCCA-1 has a good self-cleaning function. The aerogel prepared also has high elastic characteristics. Aerogel's mechanical characteristics may be significantly enhanced by the 3D nanofiber structure and bonding network, which also exhibits recoverable elasticity and flexibility [82, 83]. When NCCA-1 is compressed slightly to a flat state and released, the aerogel can easily return to its original form (Fig. S36), with high compressive strength and good fatigue performance.

## Conclusions

In this work, a simple self-assembly of NiCo-MOF hydrogels by water-induced self-assembly is explored to transform the conventional layered structure into nanofibrous and form NiCo/C aerogels by pyrolysis. Due to the suitable impedance matching, excellent conductive loss, dipole polarization, interfacial polarization and special 3D mesh-like fiber structure, it has good attenuation ability for incident electromagnetic waves. The results show that the *RL*_min_ of NCCA-1 is − 60.67 dB at 2.2 mm, and the EAB_max_ is 6.22 GHz at a matched thickness of 1.9 mm. In addition, the prepared aerogel has a nanofiber morphology endowed with hydrophobic, lightweight, and self-cleaning functions with strong stability. It makes up for the application of traditional absorbing materials in practice, and provides a potential reference for further research on multifunctional absorbers.

### Supplementary Information

Below is the link to the electronic supplementary material.

Supplementary file1 (PDF 2859 KB)
