# Supplementary material for: Construction of Self-Assembly Based Tunable Absorber: Lightweight, Hydrophobic and Self-Cleaning Properties
Source: Nanomicro Lett. 2023 May 28;15:137. doi: 10.1007/s40820-023-01108-3 (PMC10225461; doi:10.1007/s40820-023-01108-3)
Supplement: Supplementary file 1 — Supplementary file1 (PDF 2859 KB) [file 40820_2023_1108_MOESM1_ESM.pdf]

Supporting Information for

# Construction of Self-Assembly Based Tunable Absorber: Lightweight, Hydrophobic and Self-Cleaning Properties

Zehua Zhou[1, #], Qianqian Zhu[2, #], Yue Liu[1], Yan Zhang[1], Zirui Jia[2, *], Guanglei Wu[1, *]

[1] Institute of Materials for Energy and Environment, State Key Laboratory of Bio-fibers and Eco-textiles, College of Materials Science and Engineering, Qingdao University, Qingdao 266071, P. R. China

[2] College of Chemistry and Chemical Engineering, Qingdao University, Qingdao 266071, P. R. China

#Zehua Zhou and Qianqian Zhu contributed equally to this work

*Corresponding authors. E-mail: jiazirui@qdu.edu.cn (Z. Jia); wuguanglei@qdu.edu.cn (G. Wu)

## Supplementary Figures

Previously, we investigated the morphology, hydrophobic properties, and EMA performance of monometallic Ni/C and Co/C aerogels with different molar ratios of Ni (Co) and BTC. The results showed that changing the molar ratios of Ni (Co) and BTC had no effect on the final samples. As shown in Fig. S1 and Fig. 1c, all detected similar diffraction peaks demonstrate the successful synthesis of Ni/C and Co/C aerogels. The morphology of the precursor MOF does not change significantly with the change of the molar ratios, and all show a lamellar structure (Figs. S5a, d, S6a-c, and 7a-c). Compared with the other three ratios, Ni-MOF-2 and Co-MOF-2 have more layers and are more regular, which directly leads to longer lengths, finer diameters, and higher uniformity of the nanofibers in the aerogels synthesized after water induction (Figs. S5b, c, e, f, S6d-f, and S7d-f). With this regular structure, the hydrophobic (Figs. 7$d_1$, $d_2$, S34, and S35) and EMA performance (Figs. S25-S30) are better than the other three ratios. Since there is no hydrophilic group (Figs. S4 and 7c), they all have hydrophobic properties, with NCA-2 and CCA-2 reaching water contact angle angles of 141.7 and 142.6 (Fig. 7$d_1$, $d_2$) with excellent hydrophobic properties; while in terms of EMA performance: NCA-2 reaches -57.99 dB at 4.6 mm for $RL_{min}$ and 5.2 GHz for EAB at 2.3 mm; CCA-2 reaches -58.04 dB at 4.7 mm for $RL_{min}$ and 5.84 GHz for EAB at 2.3 mm. The other three ratios, either short in matching thickness or $RL_{min}$ or EAB, do not attenuate the electromagnetic wave well. We can also verify further from $\varepsilon'$, $\varepsilon''$, $\tan\delta_\varepsilon$, $\mu'$, $\mu''$, $\tan\delta_\mu$ (Figs. S19-S20), Raman spectrum (Figs. S3 and 1d), Col-Col plots (Figs. S22-S23 and 3$c_1$, $c_2$), impedance matching (Fig. S32), NCA-2 and CCA-2 have more abundant dielectric and magnetic losses, producing more polarization relaxation process to attenuate electromagnetic wave. Based on this, we selected NCA-2 and CCA-2 for a one-step study to replace the monometallic Co or Ni with bimetallic Co Ni to further research the effect of bimetallic synergy on the performance of MOF-based aerogels.

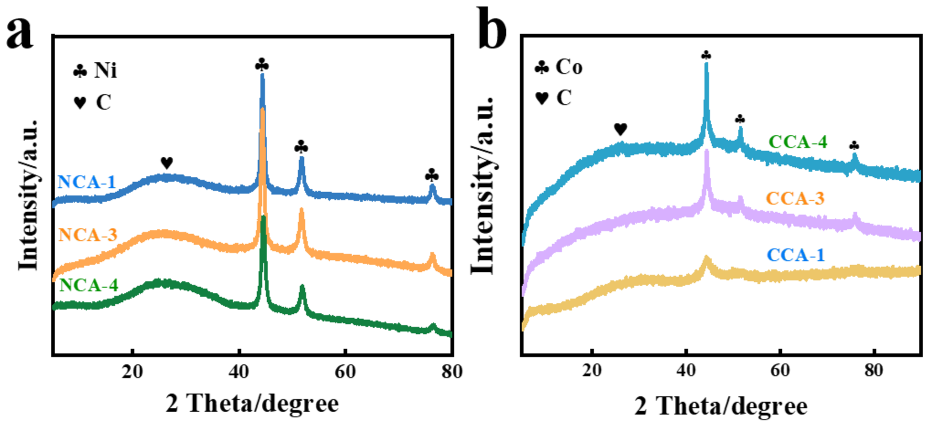


**Fig. S1** XRD pattern of **a** NCA-1, NCA-3, NCA-4 and **b** CCA-1, CCA-3, CCA-4

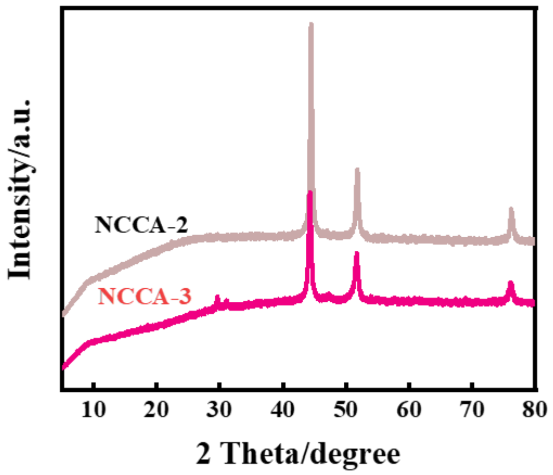


**Fig. S2** XRD pattern of NCCA-2, NCCA-3

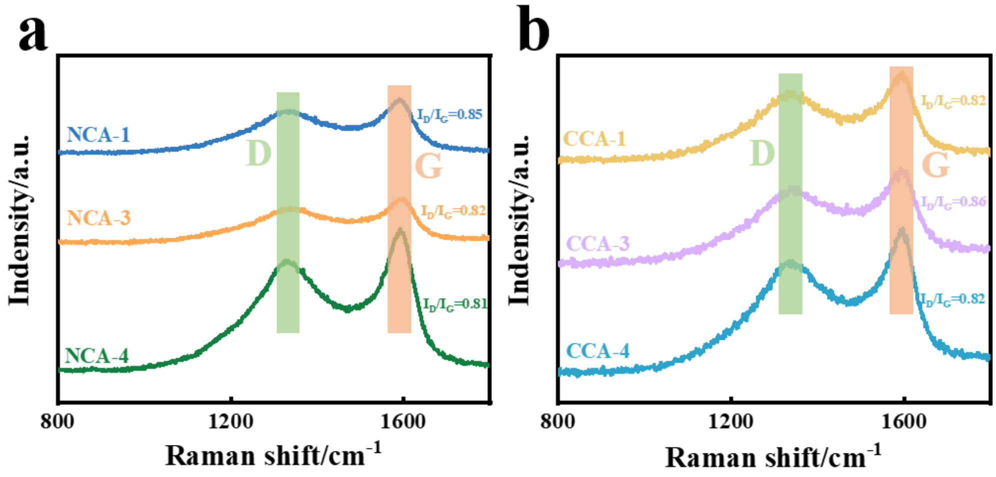


**Fig. S3 a** Raman spectra of NCA-1, NCA-3, NCA-4 and **b** CCA-1, CCA-3, CCA-4

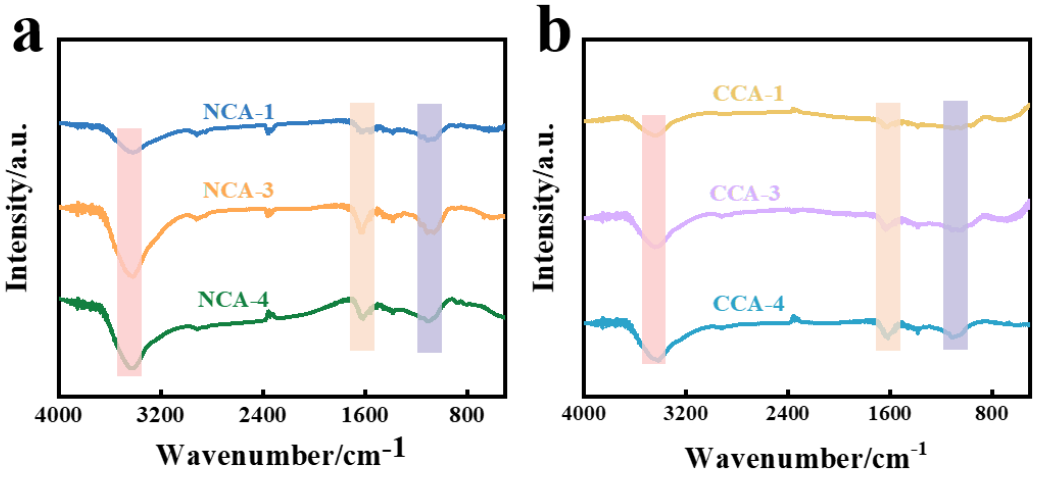


**Fig. S4** FT-IR pattern of **a** NCA-1, NCA-3, NCA-4 and **b** CCA-1, CCA-3, CCA-4

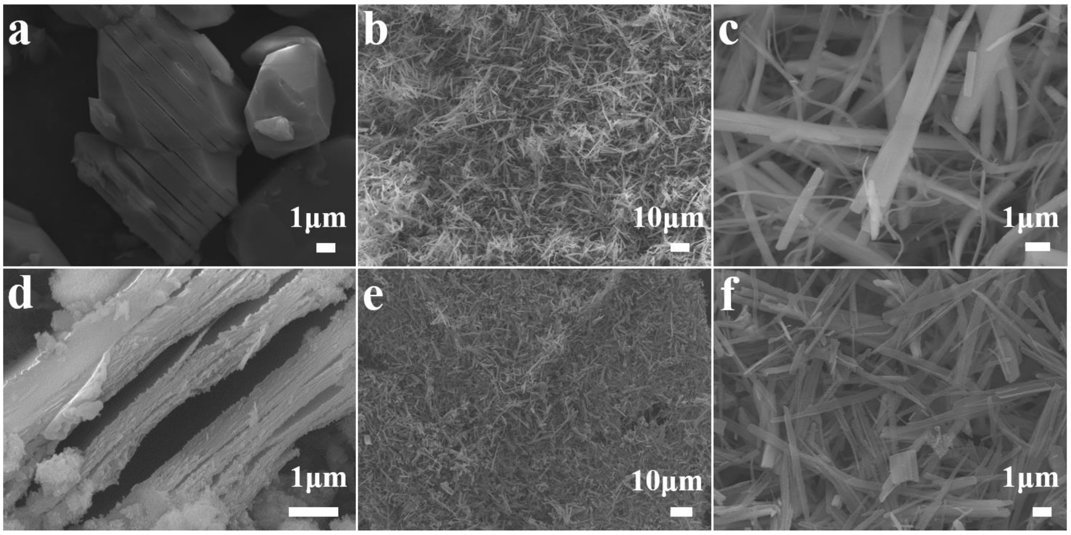


**Fig. S5** SEM images of **a** Ni-MOF-2, **b, c** NCA-2, **d** Co-MOF-2, **e, f** CCA-2

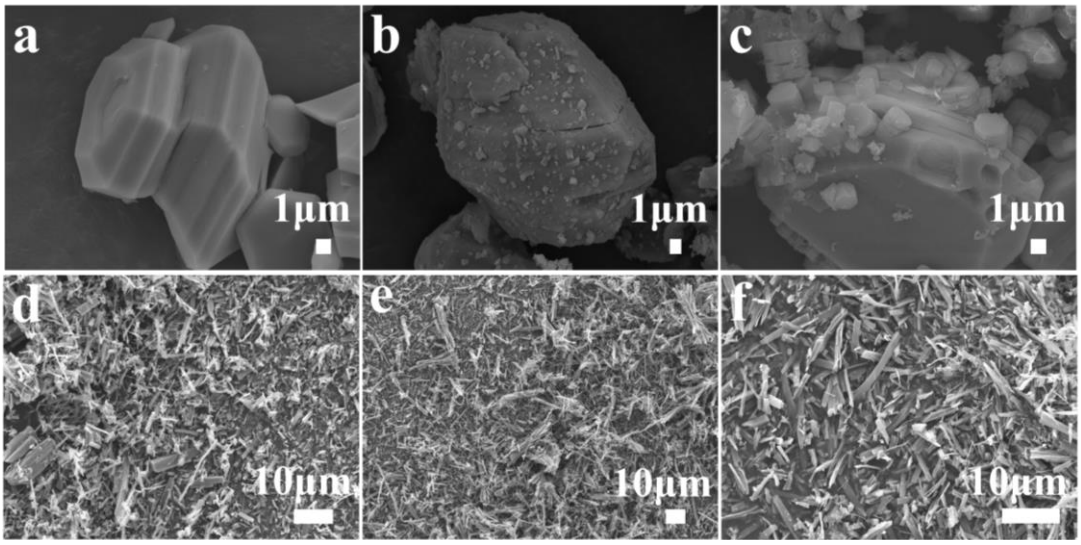


**Fig. S6** SEM images of **a** Ni-MOF-1, **b** Ni-MOF-3, **c** Ni-MOF-4, **d** NCA-1, **e** NCA-3, **f** NCA-4

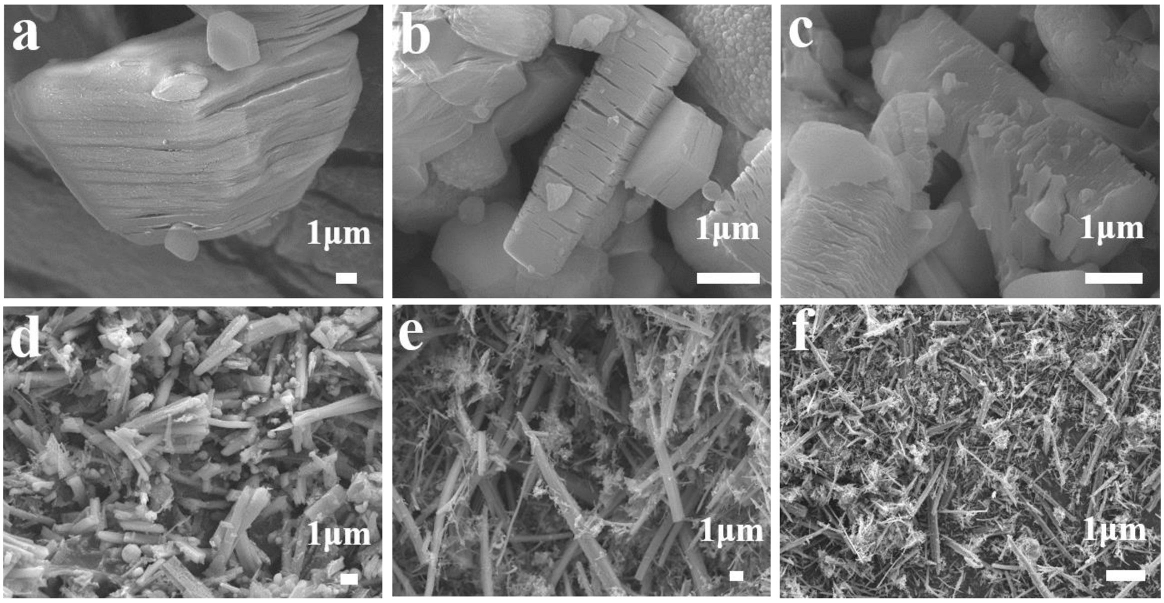


**Fig. S7** SEM images of **a** Co-MOF-1, **b** Co-MOF-3, **c** Co-MOF-4, **d** CCA-1, **e** CCA-3, **f** CCA-4

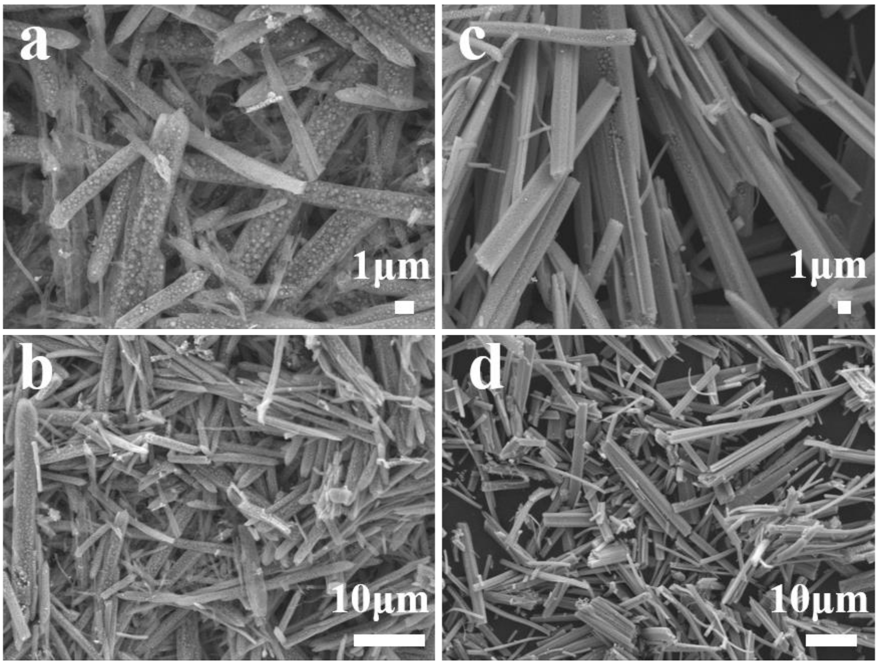


**Fig. S8** SEM images of **a, b** NCCA-2 and **c, d** NCCA-3 at different magnifications

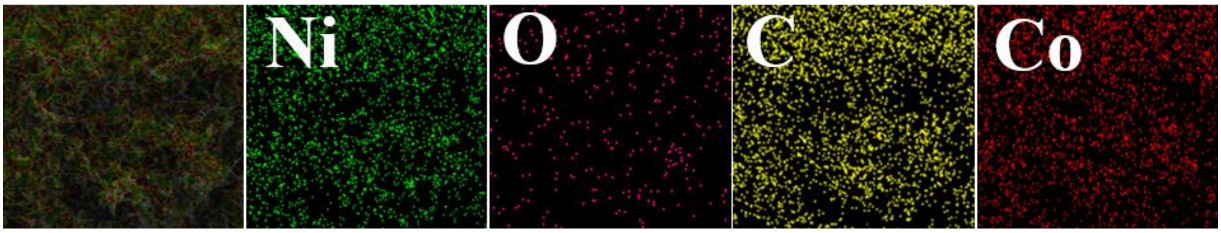


**Fig. S9** EDS image of NCCA-1

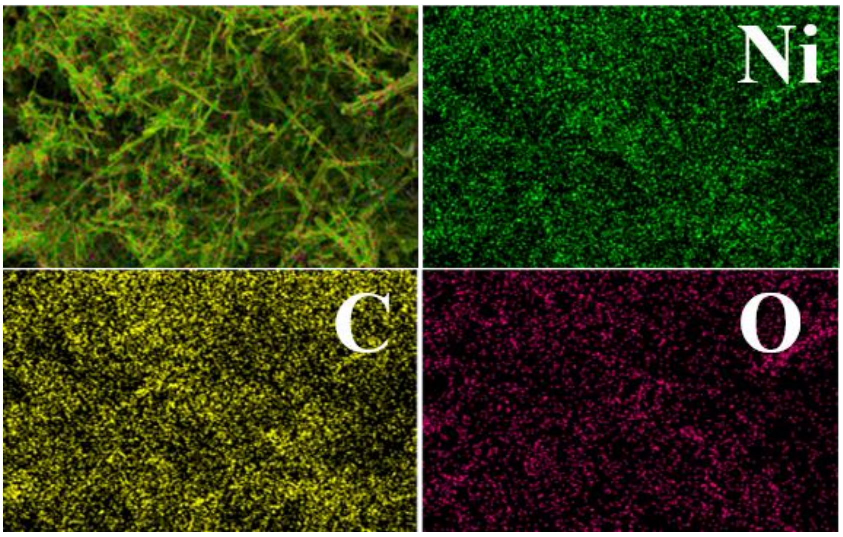


**Fig. S10** EDS image of NCA-2

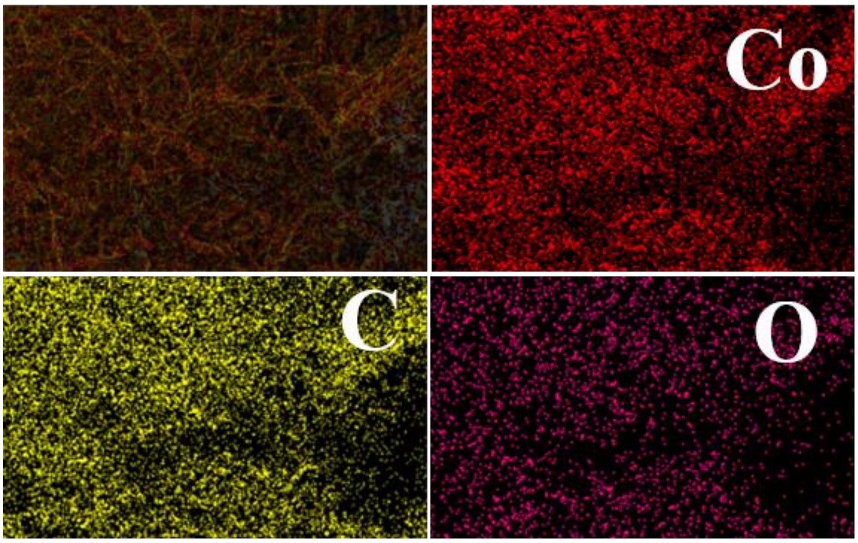


**Fig. S11** EDS image of CCA-2

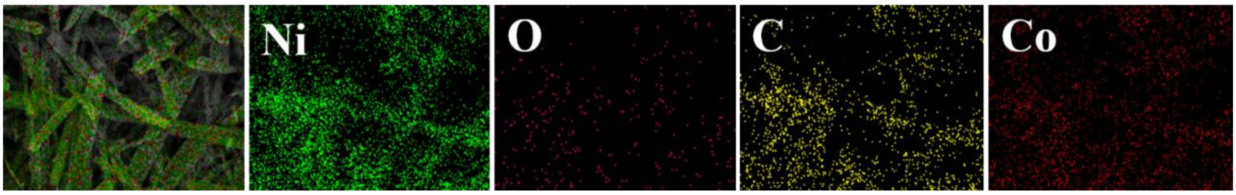


**Fig. S12** EDS image of NCCA-2

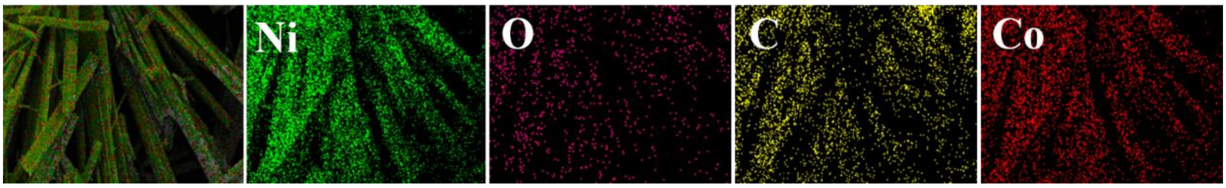


**Fig. S13** EDS image of NCCA-3

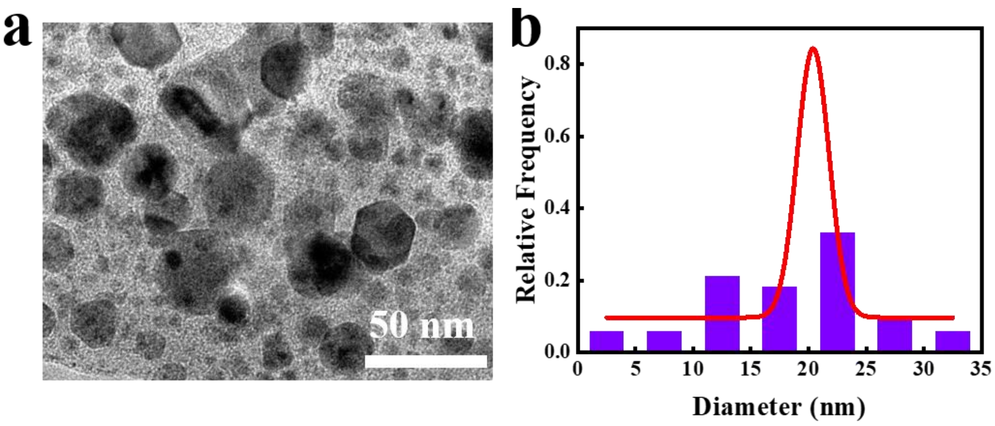


**Fig. S14 a** TEM of NCCA-1. **b** Diameter distribution of NiCo nanoparticles

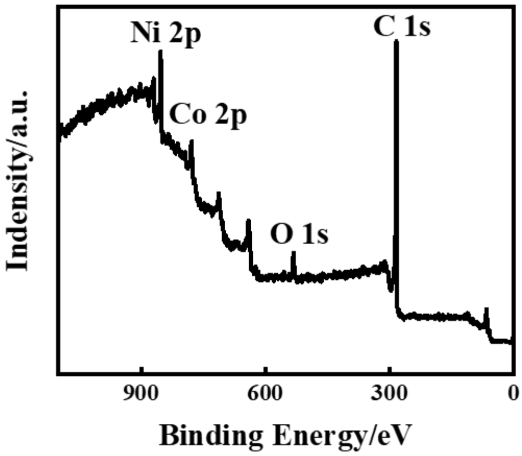


**Fig. S15** XPS survey spectra of NCCA-1

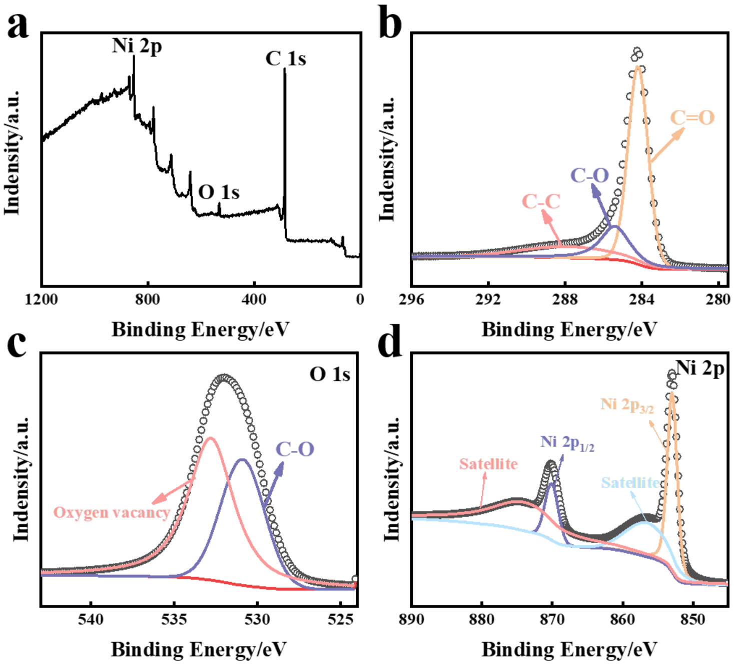


**Fig. S16 a** XPS survey spectra of NCA-2. **b** C 1s spectra, **c** O 1s spectra, **d** Ni 2p of the NCA-2

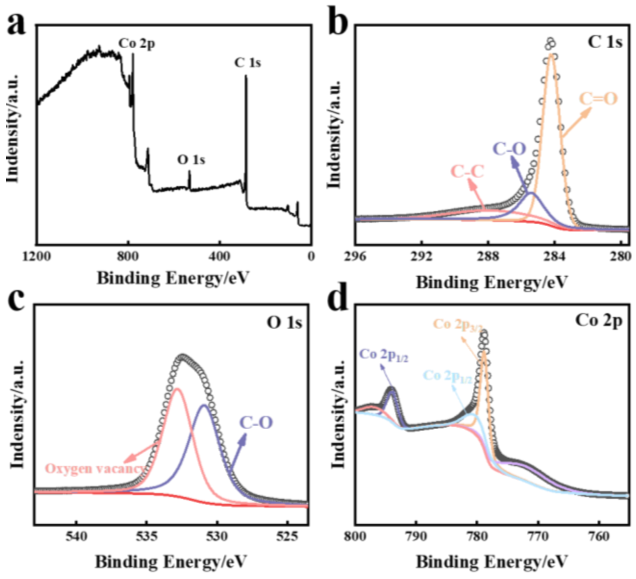


**Fig. S17 a** XPS survey spectra of CCA-2. **b** C 1s spectra, **c** O 1s spectra, **d** Ni 2p of the CCA-2

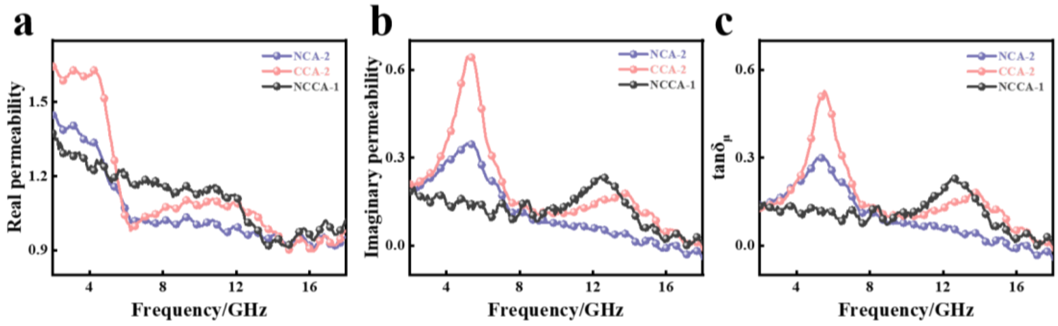


**Fig. S18** Frequency dependence of **a** μ′, **b** μ″ and **c** $\tan\delta_\mu$ of NCA-2, CCA-2, NCCA-1

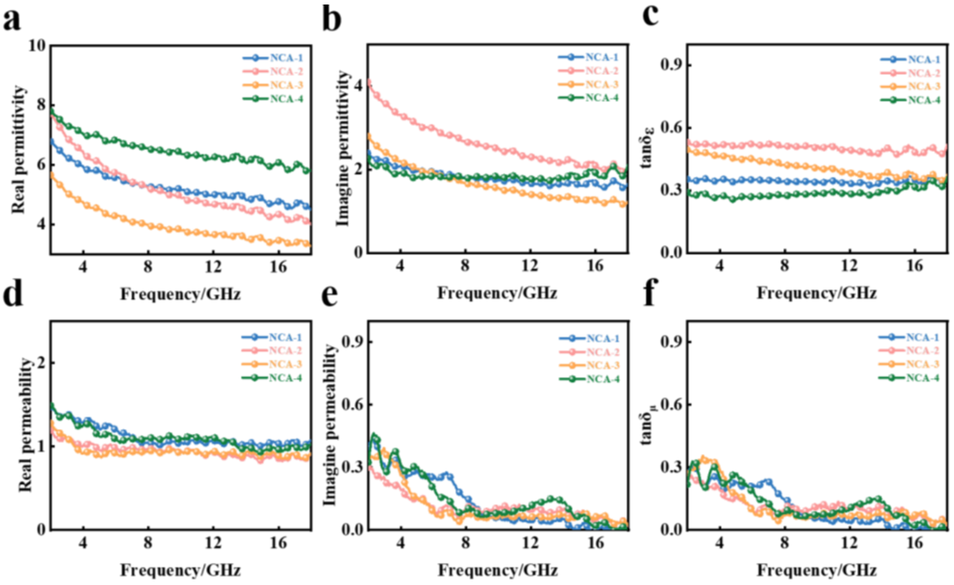


**Fig. S19** Frequency dependence of **a** ε′, **b** ε″, **c** $\tan\delta_\varepsilon$, **d** μ′, **e** μ″ and **f** $\tan\delta_\mu$ of NCA-1, NCA-2, NCA-3, NCA-4

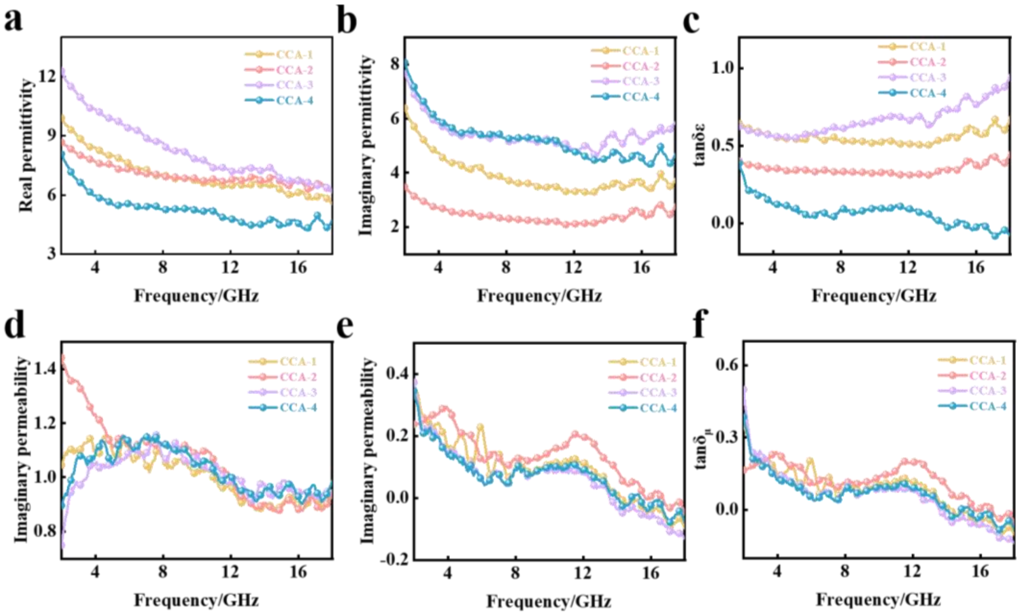


**Fig. S20** Frequency dependence of **a** ε′, **b** ε″, **c** tan$\delta_\varepsilon$, **d** μ′, **e** μ″ and **f** tan$\delta_\mu$ of CCA-1, CCA-2, CCA-3, CCA-4

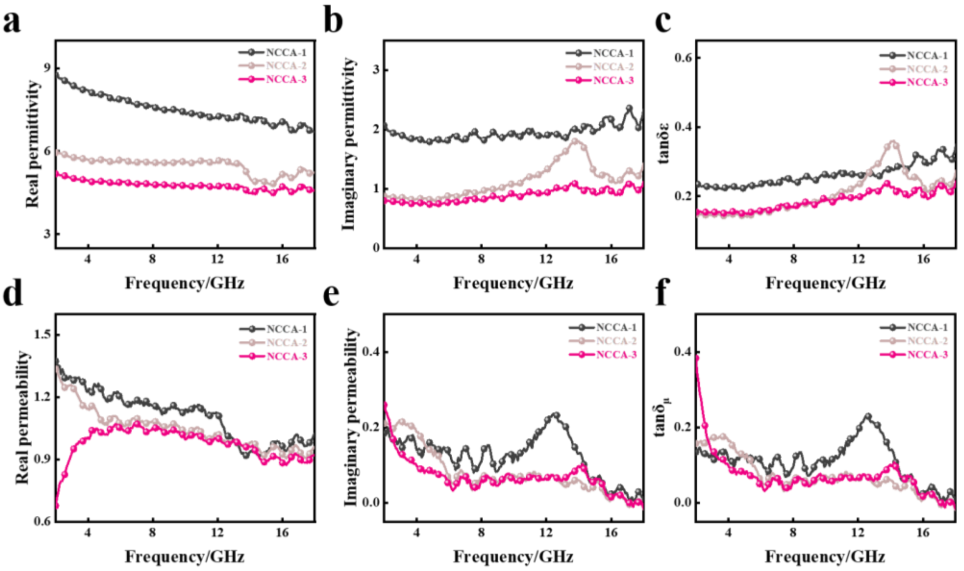


**Fig. S21** Frequency dependence of **a** ε′, **b** ε″, **c** tan$\delta_\varepsilon$, **d** μ′, **e** μ″ and **f** tan$\delta_\mu$ of NCCA-1, NCCA-2, NCCA-3

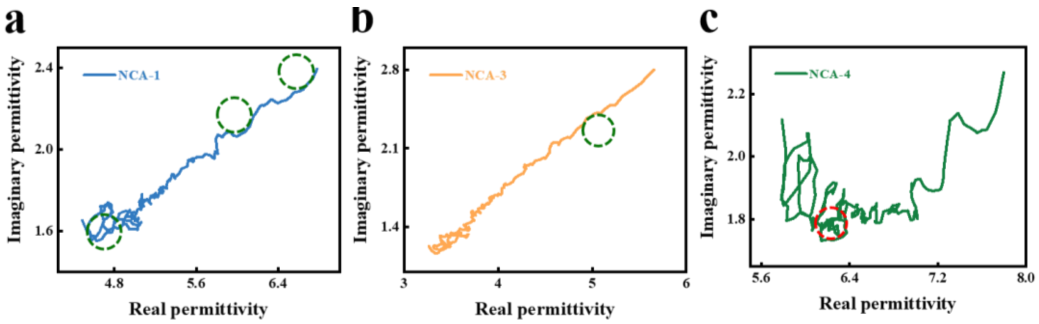


**Fig. S22** Cole–Cole plots of **a** NCA-1, **b** NCA-3, **c** NCA-4

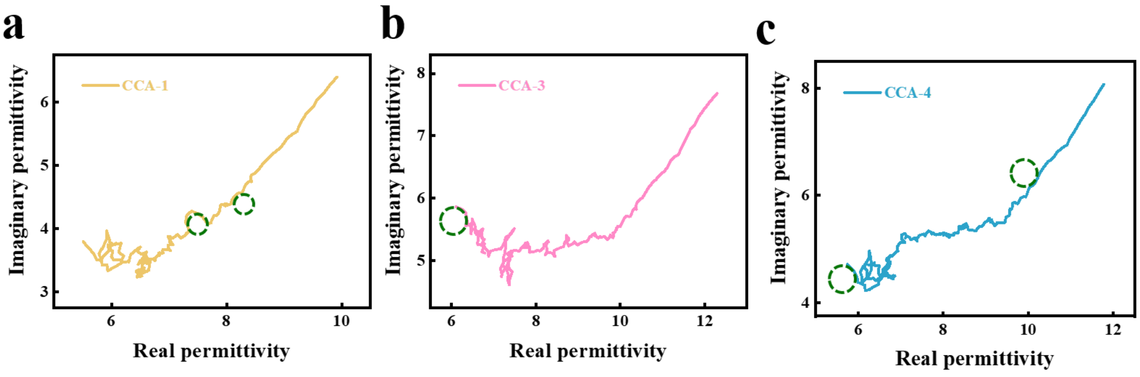


**Fig. S23** Cole–Cole plots of **a** CCA-1, **b** CCA-3, **c** CCA-4

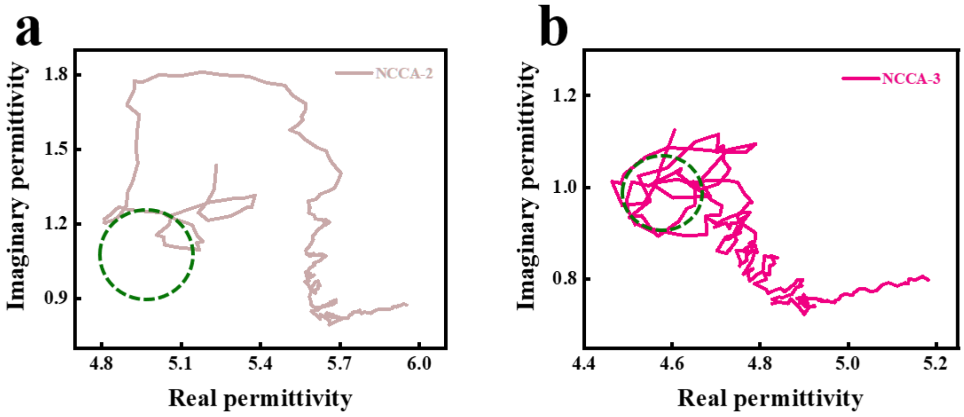


**Fig. S24** Cole–Cole plots of **a** NCCA-2, **b** NCCA-3

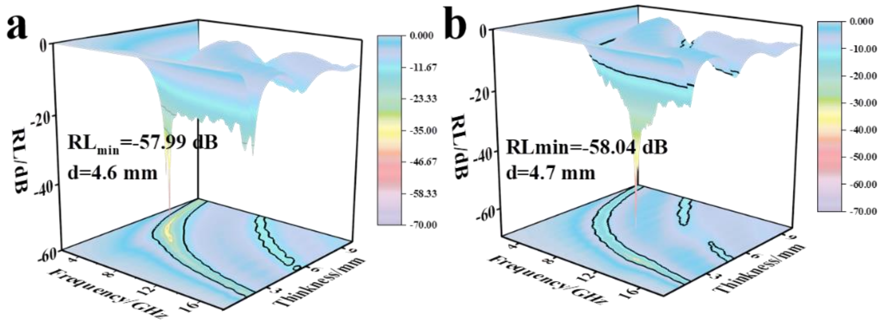


**Fig. S25** 3D RL diagrams of **a** NCA-2 and **b** CCA-2

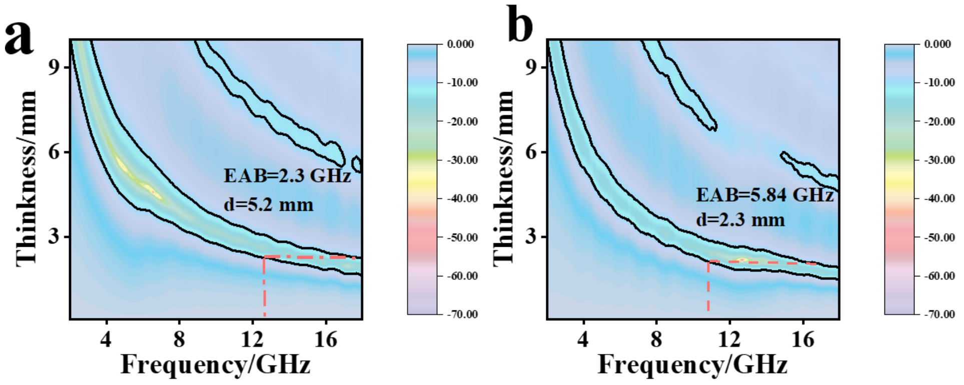


**Fig. S26** 2D RL diagrams of **a** NCA-2 and **b** CCA-2

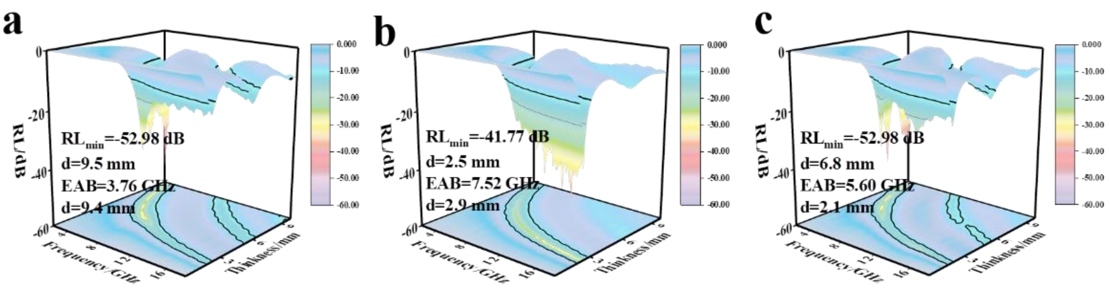


**Fig. S27** 3D RL diagrams of **a** NCA-1, **b** NCA-3 and **c** NCA-4

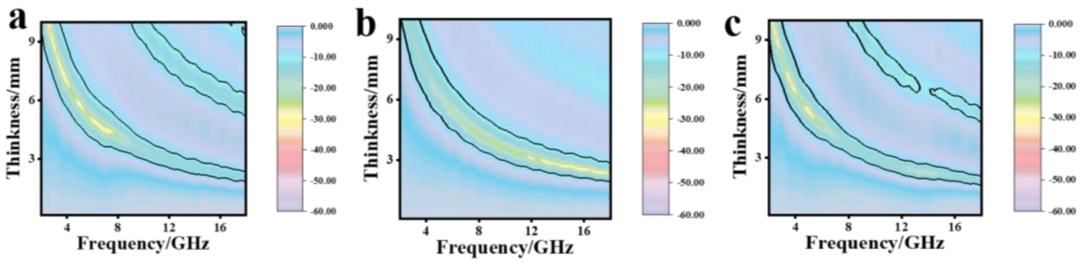


**Fig. S28** 2D RL diagrams of **a** NCA-1, **b** NCA-3 and **c** NCA-4

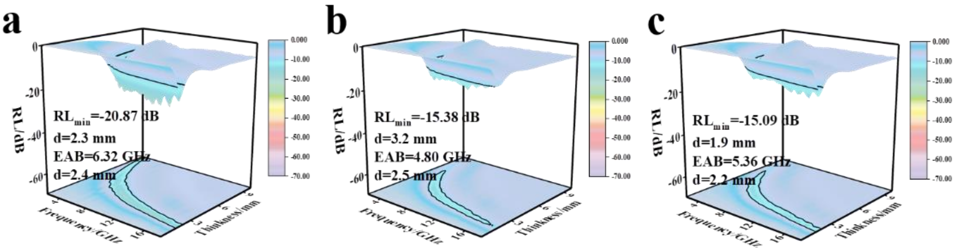


**Fig. S29** 3D RL diagrams of **a** CCA-1, **b** CCA-3 and **c** CCA-4

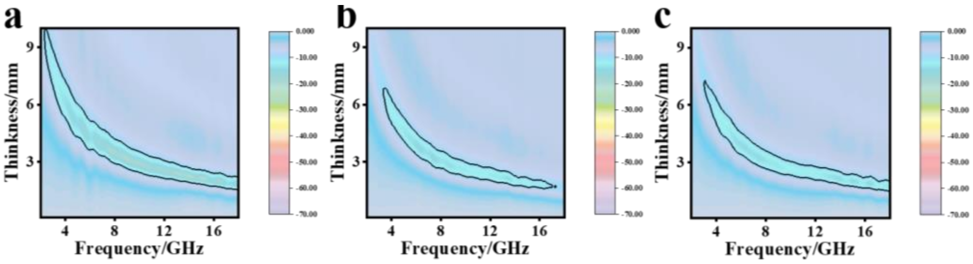


**Fig. S30** 3D RL diagrams of **a** CCA-1, **b** CCA-3 and **c** CCA-4

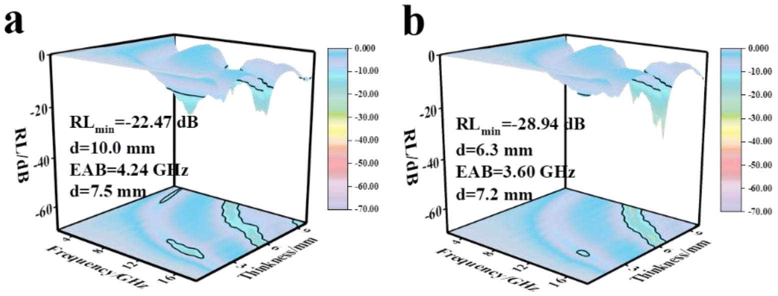


**Fig. S31** 3D RL diagrams of **a** NCCA-2, **b** NCCA-3

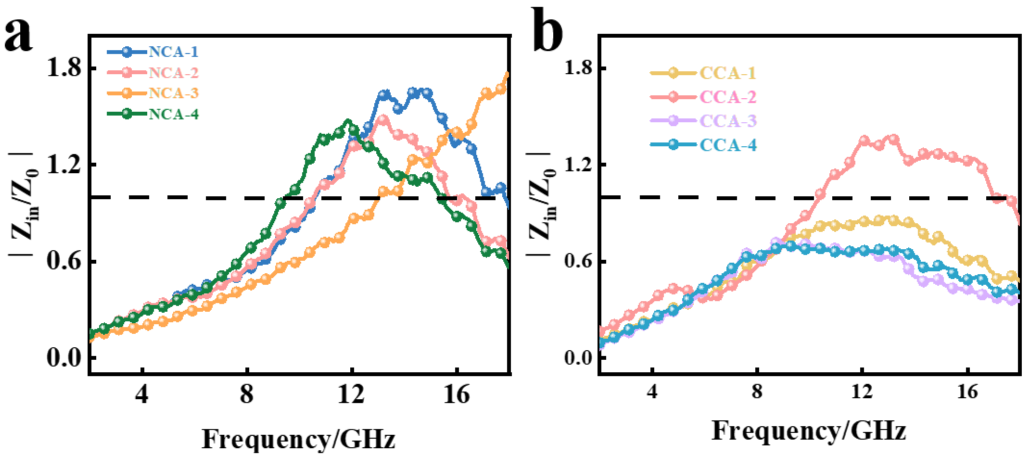


**Fig. S32** Impedance matching of **a** NCA-1, NCA-2, NCA-3, NCA-4 and **b** CCA-1, CCA-2, CCA-3, CCA-4

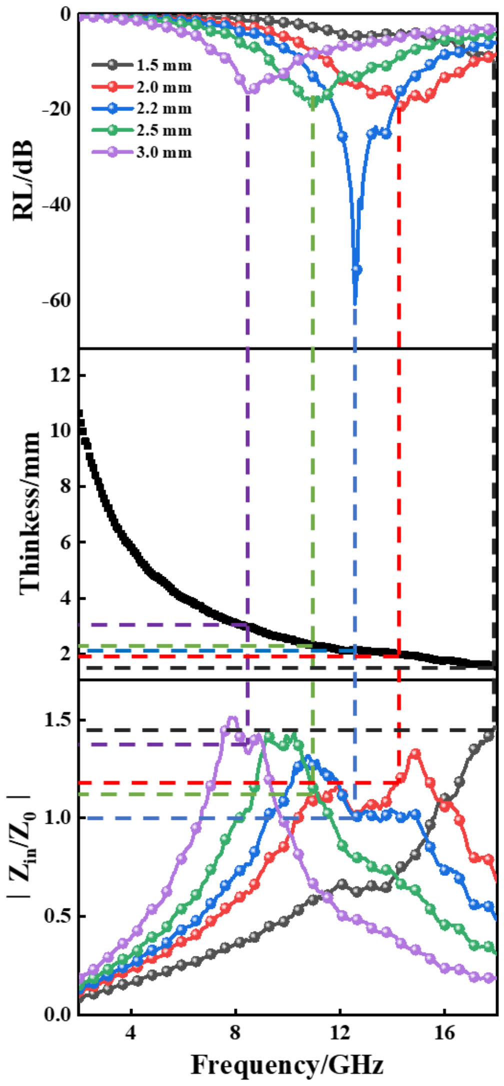


**Fig. S33** Dependence of λ/4 matching thickness vs RL peak vs $|Z_{in}/Z_0|$ plots of NCCA-1

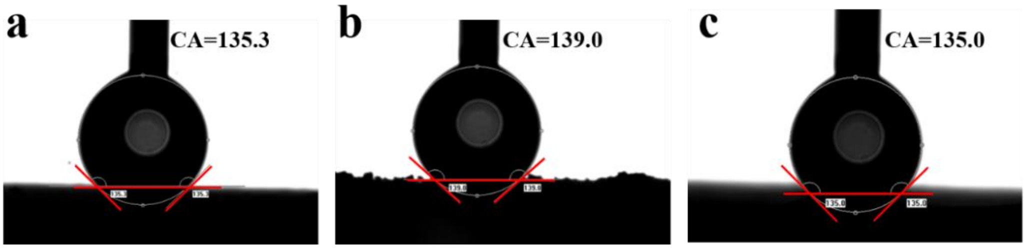


**Fig. S34** Water contact angle of **a** NCA-1, **b** NCA-3, **c** NCA-4

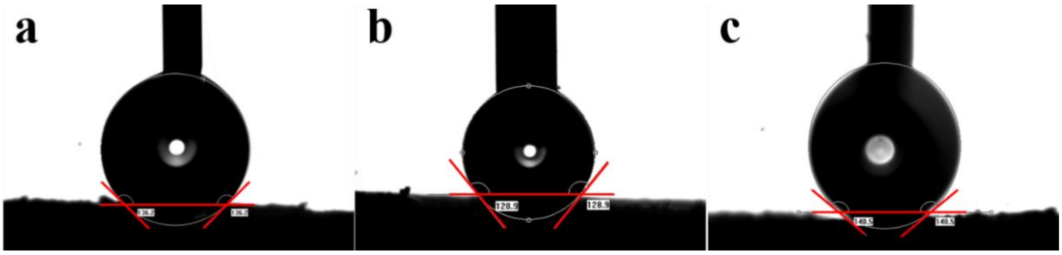


**Fig. S35** Water contact angle of **a** CCA-1, **b** CCA-3, **c** CCA-4

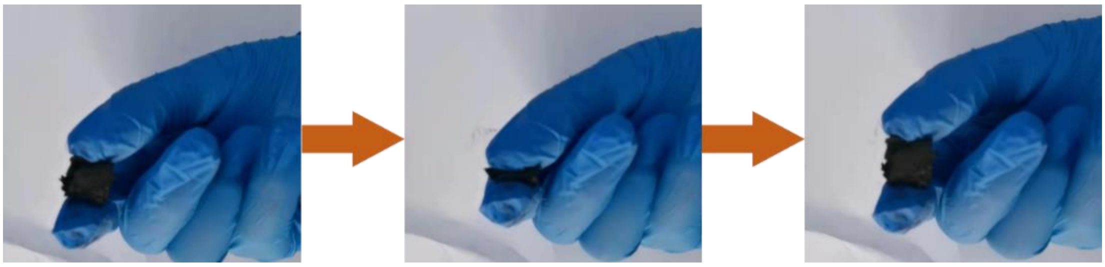

**Fig. S36** Elasticity test of NCCA-1
